# Innovative solutions for lossy nonlinear transmission lines model using a modified extended mapping approach with fractional effects

**DOI:** 10.1038/s41598-026-35652-w

**Published:** 2026-03-09

**Authors:** Hisham H. Hussein, Wassim Alexan, Shaimaa A. Kandil

**Affiliations:** 1 School of Mathematical and Computational Sciences, University of Prince Edward Island (UPEI), Cairo Campus, The New Administrative Capital, Egypt; 2 Communications Department, Faculty of IET, The German University in Cairo (GUC), Cairo, Egypt; 3 Department of Electrical Power and Machine Engineering, Faculty of Engineering, Capital University (formerly Helwan University), Cairo, Egypt

**Keywords:** Lossy transmission lines, Modified extended mapping method, Soliton solutions, Conformable fractional derivatives, Nonlinear partial differential equations, Engineering, Mathematics and computing, Optics and photonics, Physics

## Abstract

This study investigates soliton solutions of the lossy nonlinear electrical transmission line (Loss-NLETL) model using the Modified Extended Mapping (Mod-EM) technique. The model incorporates the effect of a conformable fractional derivative (Con-FD) with respect to the spatial variable $x$, enabling a more generalized and exact depiction of the system’s behavior. A set of exact analytical solutions has been obtained, including composite hyperbolic-type solutions, trigonometric periodic waves, singular periodic wave solutions, dark soliton solutions, exponential traveling wave solutions, hyperbolic soliton solutions, singular hyperbolic waveforms, mixed-type soliton structures involving kink and rational hyperbolic components, and Jacobi elliptic wave solutions. Several of these solutions are novel and extend the existing literature. To illustrate the physical behavior and structural diversity of the solutions, 2D, 3D, and density plots have been generated. Furthermore, a parametric analysis has been conducted to explore the influence of the variation in ${\beta }_{1}$​, a key parameter affecting the spatial evolution of the waveforms. The results demonstrate the effectiveness of the method in generating diverse soliton structures in complex nonlinear systems, with potential implications for applied physics and electrical engineering applications.

## Introduction

Transmission lines are fundamental components in electrical engineering, forming the backbone for transmitting both power and signals across a wide range of systems^1,2^. Beyond their traditional roles, their importance has grown significantly in modern electronics, particularly due to the emergence of nonlinear behaviors when interacting with semiconductor devices such as varactors^3,4^. These complex behaviors are effectively characterized by the Loss-NLETL model, which accounts for phenomena such as voltage-dependent capacitance and dispersion effects^5,6^. As a result, in various electrical applications, these models are invaluable for achieving precise pulse shaping, enabling efficient high-speed switching, and ensuring optimal energy transfer within advanced power electronic circuits^7^.

Furthermore, in communication systems, nonlinear transmission lines play a pivotal role in generating and sustaining robust soliton-like pulses that preserve their shape and intensity over long propagation distances^8,9^. This property makes them particularly well-suited for critical applications such as radar, ultra-wideband communication, and high-performance antenna systems, where accurate amplification of short pulses and signal integrity are essential^10^. Recent analytical advances have substantially deepened our understanding of soliton dynamics in lossy environments, despite the challenges posed by resistance and dielectric losses. These insights further highlight the vital role of transmission lines at the intersection of power engineering and cutting-edge telecommunications technologies^1,5,8^.

Previous studies have employed a range of computational methods to investigate soliton dynamics in Loss-NLETL. For RLC transmission lines under the small amplitude and long wavelength limit, coupled Ginzburg–Landau equations were derived using the reductive perturbation approach and complex expansion. This facilitated the examination of soliton-like solutions and the instability of phase winding states^11^. Another line of research focused on discrete nonlinear transmission lines (NLTL) with varactors, where the reductive perturbation method was applied to obtain a nonlinear Schrödinger (NLS) equation with a loss term, predicting bright soliton profiles and validating these predictions through numerical simulations and experimental prototypes. The observed changes in soliton amplitude and width were consistent with theoretical expectations^12^. Further investigations employed the $G^{\prime}/G^{2}$ expansion method on ${\beta }_{j}$ derivative-based Loss-NLETL, producing diverse soliton-like pulses such as periodic, bright, and singular waves. These works emphasized the influence of fractional parameters on wave characteristics and compared the beta derivative with alternative formulations, underscoring its advantages in capturing nonlinearity and dispersion^13,14^. Moreover, reformulating the equations of Loss-NLETL into Hamiltonian form has been shown to provide clearer insights into phase behavior, chaos, and system stability. Collectively, these findings demonstrate the promise of solitons in enhancing telecommunications performance and data transfer^14^.

For a wide range of nonlinear partial differential equations (PDEs), the Mod-EM method has proven effective in identifying precise traveling-wave solutions. Recent applications, such as those involving the 3D fractional WBBM equations, have produced periodic and rational forms, as well as various soliton structures including bright, dark, and mixed solitons^15^. The method has also demonstrated strong effectiveness in addressing nonlinear wave equations such as the Gilson–Pickering equation, yielding a diverse spectrum of solutions, ranging from shock and singular waves to elliptic and periodic forms, and surpassing the solution diversity offered by several conventional approaches^16^. These findings underscore the Mod-EM method’s capacity to generate novel soliton solutions and its significant contributions to advancing the understanding of nonlinear phenomena across multiple scientific and engineering domains.

Since the seventeenth century, fractional derivatives have attracted significant scholarly attention. One particularly straightforward and local formulation, noted for its compatibility with classical calculus, is the Con-FD^17^. Traditional techniques such as the Riemann–Liouville^18^ and Caputo derivatives^19,20^ are widely used to solve fractional differential equations, but recent studies show that the Con-FD is also highly effective for nonlinear problems. Asıf Yokus et al.^21^ conducted a comparative study of the Caputo and Con-FD formulations, revealing close similarities in their behavior over narrow ranges, with only minor differences between local and nonlocal operators^22^. Owing to its analytical simplicity and its ability to capture memory effects without the complexity of nonlocal kernels, the Con-FD was selected for our investigation. This property makes it particularly well-suited for modeling the spatiotemporal dynamics of nonlinear lossy transmission lines.

Nonlinear fractional equations have been successfully addressed using the Con-FD, yielding precise soliton solutions. For example, significant wave behaviors were identified through the application of hyperbolic techniques to fractional coupled Burgers’ equations^23^. Similarly, the Con-FD has been applied to fractional stochastic models such as the Kraenkel-Manna-Merle equation, revealing diverse waveforms including periodic, kink, and anti-kink solutions. These studies highlight the role of fractional derivatives in explaining complex phenomena in ferromagnetic materials and telecommunications, while also providing comprehensive two- and three-dimensional representations^23^. Moreover, fractional models such as the time fractional Klein-Gordon and regularized long wave equations have employed the Con-FD to generate soliton solutions such as bell-shaped waves, rogue waves, and periodic structures. Collectively, these investigations not only advance the understanding of nonlinear wave propagation but also underscore the stability and practical applications of fractional derivatives in physics and engineering^24,25^.

The novelty of this work lies in the application of the Mod-EM technique to a Loss-NLETL model incorporating both spatial and temporal conformable fractional derivatives. To the best of our knowledge, this specific combination has not been previously investigated in literature. Whereas earlier studies primarily addressed classical or time-fractional cases, the influence of such fractional terms in dissipative transmission systems remains insufficiently explored. This study addresses that gap by presenting a broad class of exact soliton solutions, including hyperbolic, trigonometric, Jacobi elliptic, and mixed-type profiles. In addition, it introduces a qualitative bifurcation analysis, which reveals center–saddle dynamics through equilibrium point classification. Collectively, these contributions enrich the analytical understanding of fractional nonlinear systems and provide a strong foundation for future research in applied physics and electrical engineering.

The rest of this paper is organized as follows:

The Loss-NLETL model and its corresponding circuit equations are introduced in Sect. "Utilization of Mod–EM approach for the model". Section "Mathematical formulation" establishes the mathematical framework, with Subsection "The obtained solutions – identification and classification" defining and analyzing the Con–FD, and Subsection "Mod–EM approach" detailing the Mod-EM approach. In Sect. “Utilization of Mod–EM approach for the model”, soliton solutions for the Loss-NLETL model are derived within the Con-FD framework using the Mod-EM technique. The findings and discussion are presented in Sect. “Results and discussion”, where Subsect. “The obtained solutions – identification and classification” outlines the derived solution functions, their classifications, and key characteristics. Selected solutions are further illustrated in Subsect. “Graphical representations of the obtained solutions” through density charts, as well as 2D and 3D plots. Finally, Sect. “Conclusion” offers concluding remarks.

## Model description - lossy electrical transmission line models (Loss-NLETL)

**Fig. 1:**
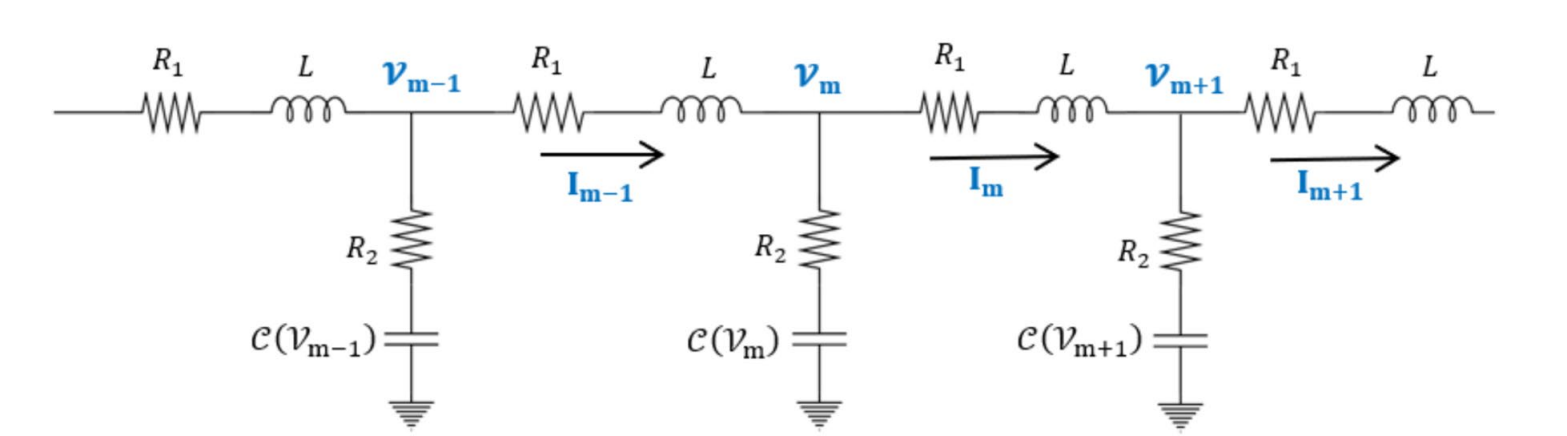
Schematic of the Loss-NLETL circuit, showing a discrete nonlinear transmission line where each segment includes an inductor $L$, series resistor ${R}_{1}$, nonlinear capacitor $c\left({U}_{i}\right)$, and shunt resistor ${R}_{2}$​.

The Lossy Nonlinear Electrical Transmission Line (Loss-NLETL) model provides a mathematical framework for analyzing the propagation of modulated waves, such as solitons, in electrical networks that exhibit both dispersive and nonlinear characteristics. This model transitions from a discrete electrical network-composed of unit cells with linear inductors, resistors, and nonlinear capacitors to a continuous system by assuming the signal wavelength is significantly larger than the inter-component spacing (Fig. 1). By applying Kirchhoff’s laws and Taylor series expansions, the discrete circuit dynamics are approximated into a continuous nonlinear partial differential equation (PDE) that governs the perturbed voltage $\mathcal{V}$ along the line. The resulting governing equation is given by^26-28^

1 $${R}_{r2}{C}_{0}{\lambda }^{2}\frac{\partial }{\partial t}\left(\frac{{\partial }^{2}\mathcal{V}}{\partial {x}^{2}}\right)+{\lambda }^{2}\left(\frac{{\partial }^{2}\mathcal{V}}{\partial {x}^{2}}\right)-{L}_{1}{C}_{0}\frac{{\partial }^{2}\mathcal{V}}{\partial {t}^{2}}+2{R}_{r1}{C}_{0}\alpha \mathcal{V}\frac{\partial \mathcal{V}}{\partial t}-{R}_{r1}{C}_{0}\frac{\partial \mathcal{V}}{\partial t}=0$$

where $\lambda$ represents the spatial separation between segments, ${C}_{0}$ is the linear capacitance per unit length, and ${L}_{1}=L/\lambda$ is the inductance per unit length. The resistance parameters ${R}_{r1}={R}_{1}/\lambda$ and ${R}_{r2}={R}_{2}/\lambda$ account for ohmic losses, while $\alpha$ denotes the nonlinearity coefficient. In this equation, the term $2{R}_{r1}{C}_{0}\alpha \mathcal{V}\frac{\partial \mathcal{V}}{\partial t}$ introduces the nonlinearity responsible for wave modulation, coupling the voltage amplitude to its rate of change, while ${R}_{r1}{C}_{0}\frac{\partial \mathcal{V}}{\partial t}$ with the negative sign serves as a linear damping term representing energy dissipation. The remaining terms describe the interplay between dispersion ${\lambda }^{2}\left(\frac{{\partial }^{2}\mathcal{V}}{\partial {x}^{2}}\right)$, fundamental wave propagation $-{L}_{1}{C}_{0}\frac{{\partial }^{2}\mathcal{V}}{\partial {t}^{2}}$, and higher-order dissipative effects.

## Mathematical formulation

To better reflect real-world behavior, fractional derivatives have been introduced in both space and time. The spatial term captures non-local effects due to voltage-dependent components and material irregularities, while the temporal term accounts for memory and dissipation over time. Together, they provide a more accurate and flexible model for how signals actually behave in nonlinear lossy transmission lines.

### Con-FD and properties

The following is the definition of the fractional-order integral differential operator that is widely recognized ^29–35^:

2 $${{}_{a}D}_{x}^{\widetilde{\beta }}=\left\{\begin{array}{cc}\frac{{d}^{\widetilde{\beta }}}{d{x}^{\widetilde{\beta }}},& f(\widetilde{\beta })>0,\\ 1,& f(\widetilde{\beta })=0,\\ {\int }_{t}^{x} d\varphi ,& f(\widetilde{\beta })<0.\end{array}\right.$$

where $f(\widetilde{\beta })$ is the real part of the fractional order $\widetilde{\beta }$ and $a$ is the lower limit of the operation. The upper limit $x$ varies with $x>a$.

Recent studies have introduced several definitions of fractional derivatives, including Con–$\mathcal{F}\mathcal{D}$, the Riemann–Liouville derivative, the modified Riemann–Liouville derivative, the Caputo derivative, the generalized Riemann–Liouville–Caputo derivative, the Caputo-Fabrizio derivative, and the Atangana-Baleanu derivative, among others. In this work, we focus on the characteristics and definitions of the Con–FD^29,30^, a simple fractional-order derivative proposed as^31-35^:

If ${\phi }_{1}:{\mathbb{R}}^{+}\to {\mathbb{R}}$ is a continuous function, then the definition of the Con–FD of order $\beta$ is written as

3 $$\frac{{\partial }^{\widetilde{\beta }}{\phi }_{1}}{\partial {t}^{\widetilde{\beta }}}=\underset{\delta \to {0}^{+}}{\mathrm{lim}} \frac{{\phi }_{1}\left(\delta {t}^{1-\widetilde{\beta }}+t\right)-{\phi }_{1}(t)}{\delta },$$

where $t$ is positive and $\delta \in (\mathrm{0,1})$. Now, considering two $\widetilde{\beta }$-differentible functions ${\phi }_{1}$ and ${\phi }_{2}$, the characteristics of the Con–$\mathcal{F}\mathcal{D}$ are as follows:

4 $$(\mathrm{i}) \frac{{\partial }^{\widetilde{\beta }}{t}^{\mathcal{a}}}{\partial {t}^{\widetilde{\beta }}}=q{t}^{\mathcal{a}-\widetilde{\beta }} \mathrm{where} \mathcal{a} \text{is a real constant},$$

5 $$(\mathrm{ii}) \frac{{\partial }^{\widetilde{\beta }}}{\partial {t}^{\widetilde{\beta }}}\left(constant\right)=0,$$

6 $$(\mathrm{iii}) \frac{{\partial }^{\widetilde{\beta }}}{\partial {t}^{\widetilde{\beta }}}\left({\mathcal{r}}_{1}{\phi }_{1}+{\mathcal{r}}_{2}{\phi }_{2}\right)={\rho }_{1}\frac{{\partial }^{\widetilde{\beta }}\phi }{\partial {t}^{\widetilde{\beta }}}+{\rho }_{2}\frac{{\partial }^{\widetilde{\beta }}{\phi }_{2}}{\partial {t}^{\widetilde{\beta }}}$$

where ${\mathcal{r}}_{1},{\mathcal{r}}_{2}$ are real constants.

7 $$\text{(iv) }\frac{{\partial }^{\widetilde{\beta }}}{\partial {t}^{\widetilde{\beta }}}\left({\phi }_{1}{\phi }_{2}\right)={\phi }_{1}\frac{{\partial }^{\widetilde{\beta }}{\phi }_{2}}{\partial {t}^{\widetilde{\beta }}}+{\phi }_{2}\frac{{\partial }^{\widetilde{\beta }}{\phi }_{1}}{\partial {t}^{\widetilde{\beta }}},$$

8 $$\text{(v) }\frac{{\partial }^{\widetilde{\beta }}}{\partial {t}^{\widetilde{\beta }}}\left(\frac{{\phi }_{1}}{{\phi }_{2}}\right)=\frac{1}{{\left({\phi }_{2}\right)}^{2}}\left({\phi }_{2}\frac{{\partial }^{\widetilde{\beta }}{\phi }_{1}}{\partial {t}^{\widetilde{\beta }}}-{\phi }_{1}\frac{{\partial }^{\widetilde{\beta }}{\phi }_{2}}{\partial {t}^{\widetilde{\beta }}}\right),$$

9 $$\left(\mathrm{vi}\right)\frac{{\partial }^{\widetilde{\beta }}{\phi }_{1}}{\partial {t}^{\widetilde{\beta }}}={t}^{\left(1-\widetilde{\beta }\right)}\frac{d{\phi }_{1}}{dt}, \text{when }{\phi }_{1}\text{ is differentiable}.$$

(vii) If ${\phi }_{1}$ and ${\phi }_{2}$ are $\widetilde{\beta }$-differentiable function of $t$ in the domain $(0,\infty )$ and ${\phi }_{2}(t)\ne 0$ then,

10 $$\frac{{\partial }^{\widetilde{\beta }}}{\partial {t}^{\beta }}\left({\phi }_{1}o{\phi }_{2}\right)\left(t\right)=\left(\frac{{\partial }^{\widetilde{\beta }}{\phi }_{1}}{\partial {t}^{\widetilde{\beta }}}\right)\left({\phi }_{2}\left(t\right)\right)\left(\frac{{\partial }^{\widetilde{\beta }}{\phi }_{2}}{\partial {t}^{\beta }}\left(t\right)\right) \left({{\phi }_{2}\left(t\right)}^{\widetilde{\beta }-1}\right).$$

### Mod–EM approach

A brief discussion of the Mod–EM strategy is included in this Sect.^15,16^. A nonlinear partial differential equation (NLPDE) can be thought of as follows:

11 $$F\left(q,{q}_{x},{q}_{t},{q}_{xt},{q}_{xx},\dots \right)=0.$$

The following steps must be taken in order to answer Eq. (11) using the Mod–EM method:

Step 1: Initially, the NLPDE in Eq. (11) is transformed into an ordinary differential equation using the wave transformation that follows:

12 $$q\left(x,t\right)=q\left(\xi \right), \xi =\frac{{m}_{1}}{{\beta }_{1}}{t}^{{\beta }_{1}}-\frac{{m}_{2}}{{\beta }_{2}}{x}^{{\beta }_{2}}.$$

where $\xi$ is linearly dependent on $x$ and $t$. Then, Eq. (11) becomes:

13 $$H\left(q,{q}{\prime},{q}^{{\prime}{\prime}},{q}^{{\prime}{\prime}{\prime}},\dots .\right)=0.$$

Step (2): The solution of Eq. (13) is supposed as:

14 $$q\left(\xi \right)=\sum_{j=-N}^{N} {\rho }_{j}{W}^{j}\left(\xi \right)+\sum_{j=2}^{N} {\psi }_{j}{W}^{j-2}\left(\xi \right){W}{\prime}\left(\xi \right)+\sum_{j=-1}^{-N} {\theta }_{-j}{W}^{j}\left(\xi \right){W}{\prime}\left(\xi \right),$$

where ${\rho }_{j},{\psi }_{j}$ and ${\theta }_{-j}$ are real constants and $W(\xi )$ satisfies the following condition:

15 $${W}{\prime}\left(\xi \right)=\sqrt{\sum_{\begin{array}{c}\upsilon =0\\ \upsilon \ne 5\end{array}}^{6}{\lambda }_{\upsilon }{\left(W\left(\xi \right)\right)}^{\upsilon }}.$$

Step (3): The highest order derivatives and the highest order nonlinear terms in Eq. (1) are balanced to determine the integer $N$.

Step (4): A set of equations for ${\varrho }_{j},{\psi }_{j},{\theta }_{-j}$ can be obtained by inserting the supposed solution in (14) and Eq. (15) into Eq. (13), then equalizing the coefficients of ${W}^{{^{\prime}}j}(\xi ){W}^{i}(\xi )(j=\mathrm{0,1};i=$ $0,\pm 1,\pm 2,\dots$ ) to zero.

Step (5): The resulting system raised in step (3) is handled by the Mathematica Package. Then, the unknown constants ${\varrho }_{j},{\psi }_{j},{\theta }_{-j}$ can be determined.

Step (6): Eq. (1) can yield a variety of solutions by varying the values of ${\lambda }_{0},{\lambda }_{1},{\lambda }_{2},{\lambda }_{3},{\lambda }_{4}$ and ${\lambda }_{6}$ in Eq. (15):

Case 1: $${\lambda }_{0}={\lambda }_{1}={\lambda }_{3}={\lambda }_{6}=0$$

16 $$W(\xi )=\sqrt{-\frac{{\lambda }_{2}}{{\lambda }_{4}}}\mathrm{sech}\left(\sqrt{{\lambda }_{2}}\xi \right), {\lambda }_{2}>0,{\lambda }_{4}<0,$$

17 $$W(\xi )=\sqrt{-\frac{{\lambda }_{2}}{{\lambda }_{4}}}\mathrm{sec}\left(\sqrt{-{\lambda }_{2}}\xi \right), {\lambda }_{2}<0,{\lambda }_{4}>0,$$

18 $$W(\xi )=\sqrt{-\frac{{\lambda }_{2}}{{\lambda }_{4}}}\mathrm{csc}\left(\sqrt{-{\lambda }_{2}}\xi \right), {\lambda }_{2}<0,{\lambda }_{4}>0.$$

Case 2: $${\lambda }_{1}={\lambda }_{3}={\lambda }_{6}=0,{\lambda }_{0}={\lambda }_{2}^{2}/4{\lambda }_{4}$$

19 $$W(\xi )=\sqrt{-\frac{{\lambda }_{2}}{2{\lambda }_{4}}}\mathrm{tanh}\left(\sqrt{-\frac{{\lambda }_{2}}{2}}\xi \right), {\lambda }_{2}<0,{\lambda }_{4}>0,$$

20 $$W(\xi )=\sqrt{\frac{{\lambda }_{2}}{2{\lambda }_{4}}}\mathrm{tan}\left(\sqrt{\frac{{\lambda }_{2}}{2}}\xi \right), {\lambda }_{2}>0,{\lambda }_{4}>0.$$

Case 3: $${\lambda }_{3}={\lambda }_{4}={\lambda }_{6}=0$$

21 $$W(\xi )=\frac{{\lambda }_{1}\mathrm{sinh}\left(2\sqrt{{\lambda }_{2}}\xi \right)}{2{\lambda }_{2}}-\frac{{\lambda }_{1}}{2{\lambda }_{2}}, {\lambda }_{2}>0,{\lambda }_{0}=0,$$

22 $$W(\xi )=\frac{{\lambda }_{1}\mathrm{sin}\left(\sqrt{-{\lambda }_{2}}\xi \right)}{2{\lambda }_{2}}-\frac{{\lambda }_{1}}{2{\lambda }_{2}}, {\lambda }_{2}<0,{\lambda }_{0}=0,$$

23 $$W(\xi )=\sqrt{\frac{{\lambda }_{0}}{{\lambda }_{2}}}\mathrm{sinh}\left(\sqrt{{\lambda }_{2}}\xi \right), {\lambda }_{0}>0,{\lambda }_{2}>0,{\lambda }_{1}=0,$$

24 $$W(\xi )=\sqrt{-\frac{{\lambda }_{0}}{{\lambda }_{2}}}\mathrm{sin}\left(\sqrt{-{\lambda }_{2}}\xi \right), {\lambda }_{0}>0,{\lambda }_{2}<0,{\lambda }_{1}=0,$$

25 $$W(\xi )=\mathrm{exp}\left(\sqrt{{\lambda }_{2}}\xi \right)-\frac{{\lambda }_{1}}{2{\lambda }_{2}}, {\lambda }_{2}>0,{\lambda }_{0}=\frac{{\lambda }_{1}^{2}}{4{\lambda }_{2}}.$$

Case 4: $${\lambda }_{0}={\lambda }_{1}={\lambda }_{6}=0$$

26 $$W\left(\xi \right)=-\frac{{\lambda }_{2}\left(\mathrm{tanh}\left(\frac{1}{2}\sqrt{{\lambda }_{2}}\xi \right)+1\right)}{{\lambda }_{3}} , {\lambda }_{3}^{2}=4{\lambda }_{2}{\lambda }_{4},{\lambda }_{2}>0,$$

27 $$W\left(\xi \right)=-\frac{{\lambda }_{2}\left(\mathrm{coth}\left(\frac{1}{2}\sqrt{{\lambda }_{2}}\xi \right)+1\right)}{{\lambda }_{3}} , {\lambda }_{3}^{2}=4{\lambda }_{2}{\lambda }_{4},{\lambda }_{2}>0,$$

28 $$W\left(\xi \right)=\frac{{\lambda }_{2}{\mathrm{sech}}^{2}\left(\frac{1}{2}\sqrt{{\lambda }_{2}}\xi \right)}{2\sqrt{{\lambda }_{2}{\lambda }_{4}}\mathrm{tanh}\left(\frac{1}{2}\sqrt{{\lambda }_{2}}\xi \right)-{\lambda }_{3}} , {\lambda }_{3}^{2}\ne 4{\lambda }_{2}{\lambda }_{4},{\lambda }_{2}>0,{\lambda }_{4}>0,$$

29 $$W\left(\xi \right)=-\frac{{\lambda }_{2}{\mathrm{sec}}^{2}\left(\frac{1}{2}\sqrt{-{\lambda }_{2}}\xi \right)}{2\sqrt{-{\lambda }_{2}{\lambda }_{4}}\mathrm{tan}\left(\frac{1}{2}\sqrt{-{\lambda }_{2}}\xi \right)+{\lambda }_{3}} , {\lambda }_{3}^{2}\ne 4{\lambda }_{2}{\lambda }_{4},{\lambda }_{2}<0,{\lambda }_{4}>0.$$

Case 5: $${\lambda }_{1}={\lambda }_{3}=0$$

30 $$W\left(\xi \right)=\sqrt{\frac{2{\lambda }_{2}{\mathrm{sech}}^{2}\left(\sqrt{{\lambda }_{2}}\xi \right)}{2\sqrt{{\lambda }_{4}^{2}-4{\lambda }_{2}{\lambda }_{6}}-\left(\sqrt{{\lambda }_{4}^{2}-4{\lambda }_{2}{\lambda }_{6}}+{\lambda }_{4}\right){\mathrm{sech}}^{2}\left(\sqrt{{\lambda }_{2}}\xi \right)}},$$

31 $$W\left(\xi \right)=\sqrt{\frac{2{\lambda }_{2}{\mathrm{sec}}^{2}\left(\sqrt{-{\lambda }_{2}}\xi \right)}{2\sqrt{{\lambda }_{4}^{2}-4{\lambda }_{2}{\lambda }_{6}}-\left(\sqrt{{\lambda }_{4}^{2}-4{\lambda }_{2}{\lambda }_{6}}-{\lambda }_{4}\right){\mathrm{sec}}^{2}\left(\sqrt{-{\lambda }_{2}}\xi \right)}}.$$

Case 6: $${\lambda }_{1}={\lambda }_{3}={\lambda }_{6}=0$$

**Table:** 

No	${\lambda }_{0}$	${\lambda }_{2}$	${\lambda }_{4}$	$W(\xi )$
1	1	$-\left(1+{m}^{2}\right)$	${m}^{2}$	$cd(\xi ,m)$ or $sn(\xi ,m)$
2	${m}^{2}-1$	$-{m}^{2}+2$	− 1	$dn(\xi ,m)$
3	$-{m}^{2}$	$2{m}^{2}-1$	$-{m}^{2}+1$	$nc(\xi ,m)$
4	− 1	$-{m}^{2}+2$	${m}^{2}-1$	$nd(\xi ,m)$
5	${m}^{2}-2{m}^{3}+{m}^{4}$	$-\frac{4}{m}$	$-1+6m-{m}^{2}$	$\frac{m dn(\xi ,m) cn(\xi ,m)}{1+{m\left(sn(\xi ,m)\right)}^{2}}$
6	$\frac{1}{4}$	$\frac{1}{2}{m}^{2}-1$	$\frac{{m}^{4}}{4}$	$\frac{sn(\xi ,m)}{1+dn(\xi ,m)}$

Case 7: $${\lambda }_{0}={\lambda }_{1}={\lambda }_{2}={\lambda }_{6}=0$$

32 $$W\left(\xi \right)=\frac{4{\lambda }_{3}}{{\lambda }_{3}^{2}{\xi }^{2}-4{\lambda }_{4}}.$$

Case 8: $${\lambda }_{2}={\lambda }_{4}={\lambda }_{6}=0$$

33 $$W(\xi )=\varnothing \left(\frac{1}{2}\sqrt{{\lambda }_{3}}\xi ;-\frac{4{\lambda }_{1}}{{\lambda }_{3}},-\frac{4{\lambda }_{0}}{{\lambda }_{3}}\right), {\lambda }_{3}>0.$$

Numerous accurate solutions to Eq. (11) can be obtained by substituting the determined constants ${\varrho }_{j},{\psi }_{j},{\theta }_{-j}$ into Eq. (14).

## Utilization of Mod–EM approach for the model

By applying the Mod–EM technique to the space–time fractional differential equation, which is stated using the Con–FD, in order to evaluate wave propagation in the Loss-NLETL. This expands on our previous application of the fractional complex transform, which has benefits over traditional derivatives, particularly when modeling memory-rich and nonlinear systems that are difficult for integer-order models to express. Fractional derivatives increase the precision of fitting experimental data and simulating real-world dynamics. In light of this, we now examine the Loss-NLETL equation in its Con–FD,

34 $${R}_{r2}{\mathcal{C}}_{0}{\lambda }^{2}{D}_{t}^{{\beta }_{2}}\left({D}_{xx}^{{2\beta }_{1}}\mathcal{V}\right)+{\lambda }^{2}\left({D}_{xx}^{{2\beta }_{1}}\mathcal{V}\right)-{L}_{1}{\mathcal{C}}_{0}{D}_{tt}^{{2\beta }_{2}}\mathcal{V}+2{R}_{r1}{\mathcal{C}}_{0}\alpha \mathcal{V} {D}_{t}^{{\beta }_{2}}\mathcal{V}-{R}_{r1}{\mathcal{C}}_{0} {D}_{t}^{{\beta }_{2}}\mathcal{V}=0.$$

Here ${D}_{x}^{{\beta }_{1}}\mathcal{V}$ and ${D}_{t}^{{\beta }_{2}}\mathcal{V}$ represent the Con–FD of $\mathcal{V}$ with respect to $t$ and $x$, respectively; where ${\beta }_{1}$ and ${\beta }_{2}$ range between $0$ and $1$. when $\beta =1$ is substituted into Eq. (34), the Loss-NLETL equation with the standard derivative is returned from the CFD equation, as shown in Eq. (10). Upon applying the transformation $U(x,t)=U(\xi )$ to Eq. (34), where,

35 $$\xi =\frac{{m}_{1}}{{\beta }_{1}}{x}^{{\beta }_{1}}-\frac{{m}_{2}}{{\beta }_{2}}{t}^{{\beta }_{2}},$$

${m}_{1}$ and ${m}_{2}$ are nontrivial constants. The resulting equation is then integrated once with respect to $\xi$, and the constant term is then equated to zero. The following ordinary differential equation is obtained,

36 $${m}_{1}^{2}{m}_{2}{R}_{r2}{\mathcal{C}}_{0}{\lambda }^{2}{\mathcal{V}}^{{\prime}{\prime}}\left(\upxi \right)+\left({L}_{1}{\mathcal{C}}_{0}{m}_{2}^{2}-{m}_{1}^{2}{\lambda }^{2}\right){\mathcal{V}}{\prime}\left(\upxi \right)+{R}_{r1}{\mathcal{C}}_{0}\alpha {m}_{2} {\mathcal{V}}^{2}\left(\upxi \right)-{R}_{r1}{C}_{0}{m}_{2}\mathcal{V}\left(\upxi \right)=0.$$

Consequently, Eq. (13) can be restructured in the following manner:

37 $${b}_{1}\mathcal{V}\left(\upxi \right)+{b}_{2}{\mathcal{V}}^{2}\left(\upxi \right)+{b}_{3}{\mathcal{V}}{\prime}\left(\upxi \right)+{b}_{4}{\mathcal{V}}^{{^{\prime}}{^{\prime}}}\left(\upxi \right)=0,$$

where, $${b}_{1}=-{R}_{r1}{\mathcal{C}}_{0}{m}_{2}, {b}_{2}=-\alpha {b}_{1}={R}_{r1}{\mathcal{C}}_{0} \alpha {m}_{2}, {b}_{3}={L}_{1}{\mathcal{C}}_{0}{m}_{2}^{2}-{m}_{1}^{2}{\lambda }^{2}$$ and $${b}_{4}={m}_{1}^{2}{m}_{2}{R}_{r2}{\mathcal{C}}_{0}{\lambda }^{2}$$.

Based on Eq. (37), the balance number is determined to be $N=2$. Thus, by substituting this value of $N$ into Eq. (14), while considering the relation given in Eq. (15), yields an intermediate expression that is substituted back into Eq. (37). This leads to the general form of the solution for Eq. (34). By collecting terms according to their order, a system of twenty-five nonlinear algebraic equations is obtained. These equations are subsequently solved using Wolfram Mathematica.

## Results and discussion

### The obtained solutions – identification and classification

**Set of Solutions 1:**

$$Set 1=\left\{{\varrho }_{1}={\varrho }_{-1}={\varrho }_{-2}={\theta }_{2}=0,{\varrho }_{0}=\frac{1}{2\alpha },{\varrho }_{2}=\frac{6{b}_{4}{\gamma }_{4}}{\alpha {b}_{1}},{\theta }_{1}=\frac{\epsilon }{2\alpha \sqrt{{\gamma }_{2}}},{b}_{3}=\frac{5\epsilon {b}_{1}}{12\sqrt{{\gamma }_{2}}}\right\}$$

At ${\gamma }_{0}={\gamma }_{1}={\gamma }_{3}={\gamma }_{6}=0,$ we get

38 $${\mathcal{V}}_{1.1}\left(x,t\right)={C}_{\mathrm{1,0}}-{C}_{\mathrm{1,1}}\mathrm{tanh}\left(\sqrt{{\gamma }_{2}} \varkappa \left(x,t\right)\right)+{C}_{\mathrm{1,2}}{\mathrm{tanh}}^{2}\left(\sqrt{{\gamma }_{2}} \varkappa \left(x,t\right)\right)-\frac{ {C}_{\mathrm{1,3}} }{\sqrt{-{\gamma }_{4}}}\mathrm{sech}\left(\sqrt{{\gamma }_{2}} \varkappa \left(x,t\right)\right)\mathrm{tanh}\left(\sqrt{{\gamma }_{2}} \varkappa \left(x,t\right)\right)$$

where $${\gamma }_{2}>0$$ and $${\gamma }_{4}<0,$$

where $\varkappa \left(x,t\right)=\frac{{m}_{1}}{{\beta }_{1}}{x}^{{\beta }_{1}}-\frac{{m}_{2}}{{\beta }_{2}}{t}^{{\beta }_{2}}$. ${\mathcal{V}}_{1.1}\left(x,t\right)$ is a composite hyperbolic-type solution that generates a smooth, asymmetric voltage pulse with sharp edges and a localized peak. Electrically, this resembles a “kink” or step, like what we see during signal switching. These stable waveforms commonly appear in nonlinear transmission lines with fractional losses, where the pulse maintains its shape as it travels.

39 $${\mathcal{V}}_{1.2}\left(x,t\right)={C}_{\mathrm{1,0}}+{C}_{\mathrm{1,1}}\mathrm{tan}\left(\sqrt{-{\gamma }_{2}} \varkappa \left(x,t\right)\right)-{C}_{\mathrm{1,2}}{\mathrm{tan}}^{2}\left(\sqrt{-{\gamma }_{2}} \varkappa \left(x,t\right)\right) +\frac{{C}_{\mathrm{1,3}}}{\sqrt{{\gamma }_{4}}}\mathrm{sec}\left(\sqrt{-{\gamma }_{2}} \varkappa \left(x,t\right)\right)\mathrm{tan}\left(\sqrt{-{\gamma }_{2}} \varkappa \left(x,t\right)\right)$$

where $${\gamma }_{2}<0$$ and $${\gamma }_{4}>0,$$

with the constants

$${C}_{\mathrm{1,0}}=\frac{1}{2\alpha }-\frac{6 {b}_{4} {\gamma }_{2}}{\alpha {b}_{1}} {C}_{\mathrm{1,1}}=\pm \frac{1}{2\alpha }$$

$${C}_{\mathrm{1,2}}=\frac{6 {b}_{4} {\gamma }_{2}}{\alpha {b}_{1}} {C}_{\mathrm{1,3}}={\gamma }_{2} {\theta }_{0}$$

is a singular periodic solution featuring sharp, repeating voltage peaks with possible singularities. In electrical terms, it models burst-like oscillations or resonant spikes, which can occur in nonlinear or overdriven circuits. These patterns are relevant in systems prone to instability, threshold-triggering**,** or nonlinear resonance behaviors.

**Set of Solutions 2**

$$Set 2=\left\{{\varrho }_{0}=\frac{{b}_{1}+\epsilon \sqrt{{b}_{1}^{2}+12 {b}_{4}^{2} {\gamma }_{2}^{2}}}{2 \alpha {b}_{1}},{\varrho }_{2}=\frac{6{b}_{4}{\gamma }_{4}}{\alpha {b}_{1}},{\varrho }_{1}={\varrho }_{-1}={\varrho }_{-2}={\theta }_{0}={\theta }_{1}={\theta }_{2}={b}_{3}=0\right\}$$

At, ${\gamma }_{0}={{\gamma }_{2}}^{2}/4{\gamma }_{4};{\gamma }_{1}={\gamma }_{3}={\gamma }_{6}=0$, we get.

40 $${\mathcal{V}}_{2.1}\left(x,t\right)={\varrho }_{0}-{\varrho }_{2}{\mathrm{tanh}}^{2}\left(\sqrt{-\frac{{\gamma }_{2}}{2}}\varkappa \left(x,t\right)\right),\text{where }{\gamma }_{2}<0,$$

41 $${\mathcal{V}}_{2.2}\left(x,t\right)={\varrho }_{0}-{\varrho }_{2}{\mathrm{tan}}^{2}\left(\sqrt{\frac{{\gamma }_{2}}{2}}\varkappa \left(x,t\right)\right),\text{ where }{\gamma }_{2}>0,$$

${\mathcal{V}}_{2.1}$ (Eq. 40) represents a dark soliton, which is essentially a localized voltage dip against a steady background. This behavior is useful in circuits that handle signal suppression or logic modulation. ${\mathcal{V}}_{2.2}\left(x,t\right)$ (Eq. 41) is a singular periodic solution characterized by sharp, repeating voltage spikes, resembling resonant or unstable oscillations. Both solutions capture real-world behaviors in nonlinear systems, such as pulse shaping, threshold-triggered responses, and resonance effects in high-speed or sensitive circuit designs.

**Set of Solutions 3**

$$Set 3=\left\{\begin{array}{c}{\varrho }_{0}=\frac{1}{2\alpha },{\varrho }_{-1}=\frac{{\epsilon }_{2} {\gamma }_{1}+2{\epsilon }_{3}\sqrt{{\gamma }_{0}{\gamma }_{2}}}{4\alpha {\gamma }_{2}},{\varrho }_{-2}=\frac{{\epsilon }_{2}{\gamma }_{0}}{2\alpha {\gamma }_{2}}, {\theta }_{1}=\frac{{\epsilon }_{1}}{2\alpha \sqrt{{\gamma }_{2}}},{\theta }_{2}=-\frac{{\epsilon }_{4}\sqrt{{\gamma }_{0}}}{2\alpha {\gamma }_{2}},{b}_{1}=6{\epsilon }_{2}{b}_{4}{\gamma }_{2},\\ {b}_{3}=5{\epsilon }_{1}{b}_{4}\sqrt{{\gamma }_{2}}, {\varrho }_{1}={\varrho }_{2}={\theta }_{0}=0\end{array}\right\}$$

At ${\gamma }_{3}={\gamma }_{4}={\gamma }_{6}=0$, we get

42 $${\mathcal{V}}_{3}\left(x,t\right)=\frac{1+{\epsilon }_{1}}{2\alpha }-\frac{{\epsilon }_{2}+{\epsilon }_{3}-{\epsilon }_{4}}{2\alpha }{e}^{-\sqrt{{\gamma }_{2}} \varkappa \left(x,t\right)}+\frac{{\epsilon }_{2}}{2\alpha }{e}^{-2\sqrt{{\gamma }_{2}} \varkappa \left(x,t\right)}.$$

${\mathcal{V}}_{3}(x,t)$ (Eq. 42) represents a family of exponential traveling wave solutions, where the voltage profile decays exponentially over space and time. Depending on the combination of parameters ${\epsilon }_{1}$ to ${\epsilon }_{4}$ (each ∈ {− 1, 1}), the waveform changes in shape and amplitude, producing eight distinct solution forms as shown in Table 1. In electrical terms, these solutions resemble decaying voltage pulses on lossy transmission lines. They are useful for modeling signal fade and energy dissipation in systems defined by fractional loss and nonlinear dispersion. Such waveforms help designers predict how pulses deform under realistic propagation conditions, which is critical in high-speed communication lines, filtering applications**,** and signal integrity analysis.

**Table 1:** The parameter arrays $\left({\epsilon }_{1}, {\epsilon }_{2}, {\epsilon }_{3}, {\epsilon }_{4}\right)$ correspond to the eight different forms of the solution, where ${\epsilon }_{j}\in \left\{-1, 1\right\}$.

${\epsilon }_{1}$	${\epsilon }_{2}$	${\epsilon }_{3}$	${\epsilon }_{4}$
$-1$	$-1$	$-1$	$-1$
$-1$	$-1$	$1$	$1$
$1$	$-1$	$1$	$-1$
$1$	$-1$	$-1$	$1$
$-1$	$1$	$1$	$-1$
$-1$	$1$	$-1$	$1$
$1$	$1$	$-1$	$-1$
$1$	$1$	$1$	$1$

where each value for $j$ is corresponding to an obtained solution.

**Set of Solutions 4**

$$Set 4=\left\{\begin{array}{c}{\varrho }_{0}=\frac{1}{2\alpha }, {\varrho }_{1}={\epsilon }_{4}\frac{\left|{\gamma }_{3}+2{\epsilon }_{3}\sqrt{{\gamma }_{2}{\gamma }_{4}}\right|}{4\alpha {\gamma }_{2}}, {\varrho }_{2}=\frac{{\gamma }_{4}}{2\alpha \sqrt{{\gamma }_{2}}\left|2\sqrt{{\gamma }_{2}{\gamma }_{4}}-{\gamma }_{3}\right|}\left(2{\epsilon }_{3}\sqrt{{\gamma }_{4}}-{\epsilon }_{4}\frac{{\gamma }_{3}}{\sqrt{{\gamma }_{2}}}\right)\\ {b}_{4}=\frac{{b}_{1}}{6\sqrt{{\gamma }_{2}}\left|2\sqrt{{\gamma }_{2}{\gamma }_{4}}-{\gamma }_{3}\right|}\left(2{\epsilon }_{3}\sqrt{{\gamma }_{4}}-{\epsilon }_{4}\frac{{\gamma }_{3}}{\sqrt{{\gamma }_{2}}}\right), {\theta }_{0}={\epsilon }_{1}\frac{\sqrt{{\gamma }_{4}}}{2\alpha {\gamma }_{2}}, {\theta }_{1}=\frac{{\epsilon }_{2}}{2\alpha \sqrt{{\gamma }_{2}}}, {b}_{3}=\frac{5{\epsilon }_{2}{b}_{1}}{6\sqrt{{\gamma }_{2}}}\\ {\theta }_{2}={\varrho }_{-1}={\varrho }_{-2}=0\end{array}\right\}$$

At ${\gamma }_{0}={\gamma }_{1}={\gamma }_{6}=0$, we get

43 $${\mathcal{V}}_{4.1}\left(x,t\right)={E}_{\mathrm{1,0}}\left({\epsilon }_{1},{\epsilon }_{4}, {\epsilon }_{3}\right)+{E}_{\mathrm{1,1}}\left({\epsilon }_{4},{\epsilon }_{3}\right)\mathrm{tanh}\left(\frac{\sqrt{{\gamma }_{2}}}{2}\varkappa \left(x,t\right)\right)+{E}_{\mathrm{1,2}}\left({\epsilon }_{1},{\epsilon }_{4}, {\epsilon }_{3}\right){\mathrm{tanh}}^{2}\left(\frac{\sqrt{{\gamma }_{2}}}{2}\varkappa \left(x,t\right)\right)+\frac{{\epsilon }_{2}{\mathrm{sech}}^{2}\left(\frac{\sqrt{{\gamma }_{2}}}{2}\varkappa \left(x,t\right)\right)}{4\alpha \left(1+\mathrm{tanh}\left(\frac{\sqrt{{\gamma }_{2}}}{2}\varkappa \left(x,t\right)\right)\right)},$$

is a hyperbolic wave solution with the coefficients ${E}_{\mathrm{1,0}}\left({\epsilon }_{4},{\epsilon }_{3},{\epsilon }_{1}\right)$, ${E}_{\mathrm{1,1}}\left({\epsilon }_{4},{\epsilon }_{3}\right)$ and ${E}_{\mathrm{1,2}}\left({\epsilon }_{4},{\epsilon }_{3},{\epsilon }_{1}\right)$. This form models a steep voltage transition with localized peaks and a rational decay pattern resembling electrical behaviors such as sharp switching**,** controlled pulse steepening**,** or nonlinear edge shaping**.** It is relevant in lossy nonlinear transmission lines where voltage transitions are not purely sinusoidal, but instead exhibit rapid shifts followed by localized energy concentration**,** as seen in high-speed logic circuits or damped signal paths. The coefficients ${E}_{\mathrm{1,0}}, {E}_{\mathrm{1,1}}$ and ${E}_{\mathrm{1,2}}$ can be defined as

44 $${E}_{\mathrm{1,0}}\left({\epsilon }_{4},{\epsilon }_{3},{\epsilon }_{1}\right)=\frac{1}{2\alpha }+\frac{{\gamma }_{2}{\gamma }_{4} \left(-{\gamma }_{3}{\epsilon }_{4}+2{\epsilon }_{3}\sqrt{{\gamma }_{2}{\gamma }_{4}}\right) \left|{\gamma }_{3}+2\sqrt{{\gamma }_{2}{\gamma }_{4}}\right|}{2\alpha {\gamma }_{3}^{2}\left(-{\gamma }_{3}^{2}+4{\gamma }_{2}{\gamma }_{4}\right)} -{\epsilon }_{4}\frac{\left|{\gamma }_{3}+2{\epsilon }_{13}\sqrt{{\gamma }_{2}{\gamma }_{4}}\right|}{4 \alpha {\gamma }_{3}}-{\epsilon }_{1}\frac{\sqrt{{\gamma }_{2}{\gamma }_{4}}}{4\alpha {\gamma }_{3}},$$

45 $${E}_{\mathrm{1,1}}\left({\epsilon }_{4},{\epsilon }_{3}\right)=\frac{{\gamma }_{2}{\gamma }_{4} \left(-{\gamma }_{3}{\epsilon }_{4}+2{\epsilon }_{13}\sqrt{{\gamma }_{2}{\gamma }_{4}}\right) \left|{\gamma }_{3}+2\sqrt{{\gamma }_{2}{\gamma }_{4}}\right| }{\alpha {\gamma }_{3}^{2}\left(-{\gamma }_{3}^{2}+4{\gamma }_{2}{\gamma }_{4}\right)} -\frac{{\epsilon }_{4} {\epsilon }_{3} \left|{\gamma }_{3}+2\sqrt{{\gamma }_{2}{\gamma }_{4}}\right|}{4 \alpha {\gamma }_{3}},$$

and

46 $${E}_{\mathrm{1,2}}\left({\epsilon }_{4},{\epsilon }_{3},{\epsilon }_{1}\right)=\frac{{\gamma }_{2}{\gamma }_{4}\left(-{\gamma }_{3}{\epsilon }_{4}+2{\epsilon }_{3}\sqrt{{\gamma }_{2}{\gamma }_{4}}\right)\left|{\gamma }_{3}+2\sqrt{{\gamma }_{2}{\gamma }_{4}}\right|}{2\alpha {\gamma }_{3}^{2}\left(-{\gamma }_{3}^{2}+4{\gamma }_{2}{\gamma }_{4}\right)}+\frac{{\epsilon }_{1}\sqrt{{\gamma }_{2}{\gamma }_{4}}}{4\alpha {\gamma }_{3}}.$$

Also, we have the solution

47 $${\mathcal{V}}_{4.2}\left(x,t\right)={E}_{\mathrm{2,0}}\left({\epsilon }_{4},{\epsilon }_{3},{\epsilon }_{1}\right)+{E}_{\mathrm{2,1}}\left({\epsilon }_{4},{\epsilon }_{3}\right)\mathrm{coth}\left(\frac{\sqrt{{\gamma }_{2}}}{2}\varkappa \left(x,t\right)\right) +{E}_{\mathrm{2,2}}\left({\epsilon }_{4},{\epsilon }_{3},{\epsilon }_{1}\right){\mathrm{coth}}^{2}\left(\frac{\sqrt{{\gamma }_{2}}}{2}\varkappa \left(x,t\right)\right)+\frac{{\epsilon }_{2}}{4\alpha }\frac{{\mathrm{csch}}^{2}\left(\frac{\sqrt{{\gamma }_{2}}}{2}\varkappa \left(x,t\right)\right)}{4\alpha \left(1+\mathrm{coth}\left(\frac{\sqrt{{\gamma }_{2}}}{2}\varkappa \left(x,t\right)\right)\right)},$$

is a singular hyperbolic soliton solution with rational decay. Electrically, it models sharp, high-amplitude voltage spikes followed by rapid decay, resembling transient bursts or overshoots often seen in nonlinear or overdriven circuits. This waveform is useful for representing behaviors such as resonant switching**,** threshold-triggered discharges, or energy localization with fast dissipation common in systems with strong nonlinearity. The accompanying coefficients are defined as:

48 $${E}_{\mathrm{2,0}}\left({\epsilon }_{4},{\epsilon }_{3},{\epsilon }_{1}\right)=\frac{1}{2\alpha }-{\epsilon }_{4}\frac{\left|{\gamma }_{3}+2\sqrt{{\gamma }_{2}{\gamma }_{4}}\right|{\gamma }_{2}{\gamma }_{4}}{2\alpha {\gamma }_{3}\left(-{\gamma }_{3}^{2}+4{\gamma }_{2}{\gamma }_{4}\right)}-{\epsilon }_{4}\frac{\left|{\gamma }_{3}+2{\epsilon }_{43}\sqrt{{\gamma }_{2}{\gamma }_{4}}\right|}{4\alpha {\gamma }_{3}} +{\epsilon }_{3}\frac{\left|{\gamma }_{3}+2\sqrt{{\gamma }_{2}{\gamma }_{4}}\right| {\left({\gamma }_{2}{\gamma }_{4}\right)}^{1.5}}{\alpha {\gamma }_{3}^{2}\left(-{\gamma }_{3}^{2}+4{\gamma }_{2}{\gamma }_{4}\right)}-{\epsilon }_{1}\frac{\sqrt{{\gamma }_{2}{\gamma }_{4}}}{4\alpha {\gamma }_{3}},$$

49 $${E}_{\mathrm{2,1}}\left({\epsilon }_{4},{\epsilon }_{3}\right)=-{\epsilon }_{4}\frac{ {\gamma }_{2}{\gamma }_{4} \left|{\gamma }_{3}+2\sqrt{{\gamma }_{2}{\gamma }_{4}}\right| }{\alpha {\gamma }_{3}\left(-{\gamma }_{3}^{2}+4{\gamma }_{2}{\gamma }_{4}\right)}+{\epsilon }_{3}\frac{2{\left({\gamma }_{2}{\gamma }_{4}\right)}^{1.5}\left|{\gamma }_{3}+2\sqrt{{\gamma }_{2}{\gamma }_{4}}\right| }{\alpha {\gamma }_{3}^{2}\left(-{\gamma }_{3}^{2}+4{\gamma }_{2}{\gamma }_{4}\right)} -{\epsilon }_{4}\frac{\left|{\gamma }_{3}+2{\epsilon }_{32}\sqrt{{\gamma }_{2}{\gamma }_{4}}\right|}{4\alpha {\gamma }_{3}},$$

and

50 $${E}_{\mathrm{2,2}}\left({\epsilon }_{4},{\epsilon }_{3},{\epsilon }_{1}\right)=-{\epsilon }_{4}\frac{ {\gamma }_{2}{\gamma }_{4} \left|{\gamma }_{3}+2\sqrt{{\gamma }_{2}{\gamma }_{4}}\right| }{2\alpha {\gamma }_{3}\left(-{\gamma }_{3}^{2}+4{\gamma }_{2}{\gamma }_{4}\right)}+{\epsilon }_{3}\frac{ {\left({\gamma }_{2}{\gamma }_{4}\right)}^{1.5}\left|{\gamma }_{3}+2\sqrt{{\gamma }_{2}{\gamma }_{4}}\right| }{\alpha {\gamma }_{3}^{2}\left(-{\gamma }_{3}^{2}+4{\gamma }_{2}{\gamma }_{4}\right)}+{\epsilon }_{1}\frac{ \sqrt{{\gamma }_{2}{\gamma }_{4}}}{4\alpha {\gamma }_{3}}.$$

51 $$\begin{gathered} {\mathcal{V}}_{{4.3}} \left( {x,t} \right) = \frac{1}{{2\alpha }} + \frac{{E_{{4,0}} \left( { \in _{4} , \in _{3} } \right)}}{{2\sqrt {\gamma _{2} \gamma _{4} } \tanh \left( {0.5\sqrt {\gamma _{2} } \left( {x,t} \right)} \right) - \gamma _{3} }} + E_{{4,1}} \left( { \in _{1} , \in _{2} } \right)\frac{{~\tanh \left( {\frac{{\sqrt {\gamma _{2} } }}{2}\left( {x,t} \right)} \right)}}{{2\sqrt {\gamma _{2} \gamma _{4} } \tanh \left( {0.5\sqrt {\gamma _{2} } \left( {x,t} \right)} \right) - \gamma _{3} }} \hfill \\ + \frac{{E_{{4,2}} \left( { \in _{4} , \in _{3} , \in _{1} } \right)~\tanh ^{2} \left( {\frac{{\sqrt {\gamma _{2} } }}{2}\left( {x,t} \right)} \right)}}{{2\sqrt {\gamma _{2} \gamma _{4} } \tanh \left( {0.5\sqrt {\gamma _{2} } \left( {x,t} \right)} \right) - \gamma _{3} }} + \frac{{E_{{4,3}} \left( { \in _{1} } \right)\tanh ^{3} \left( {\frac{{\sqrt {\gamma _{2} } }}{2}\left( {x,t} \right)} \right)}}{{2\sqrt {\gamma _{2} \gamma _{4} } \tanh \left( {0.5\sqrt {\gamma _{2} } \left( {x,t} \right)} \right) - \gamma _{3} }} \hfill \\ + \frac{{E_{{4,4}} \left( { \in _{4} , \in _{3} , \in _{1} , \in _{2} } \right)}}{{\left( {2\sqrt {\gamma _{2} \gamma _{4} } \tanh \left( {0.5\sqrt {\gamma _{2} } \left( {x,t} \right)} \right) - \gamma _{3} } \right)^{2} }}\left[ {1 - \frac{1}{2}\tanh ^{2} \left( {\frac{{\sqrt {\gamma _{2} } }}{2}\left( {x,t} \right)} \right) + \tanh ^{4} \left( {\frac{{\sqrt {\gamma _{2} } }}{2}\left( {x,t} \right)} \right)} \right] \hfill \\ + E_{{4,5}} \left( { \in _{2} } \right)\frac{{{\mathrm{sech}}^{2} \left( {\frac{{\sqrt {\gamma _{2} } }}{2}\left( {x,t} \right)} \right){\mathrm{tanh}}\left( {\frac{{\sqrt {\gamma _{2} } }}{2}\left( {x,t} \right)} \right)}}{{\left( {2\sqrt {\gamma _{2} \gamma _{4} } \tanh \left( {0.5\sqrt {\gamma _{2} } \left( {x,t} \right)} \right) - \gamma _{3} } \right)^{2} }} - \in _{2} \frac{{\gamma _{3} \sqrt {\gamma _{2} \gamma _{4} } }}{{2\alpha }}\frac{{\tanh ^{4} \left( {\frac{{\sqrt {\gamma _{2} } }}{2}\left( {x,t} \right)} \right)}}{{\left( {2\sqrt {\gamma _{2} \gamma _{4} } {\mathrm{~tanh}}\left( {0.5\sqrt {\gamma _{2} } \left( {x,t} \right)} \right) - \gamma _{3} } \right)^{2} }}, \hfill \\ \end{gathered}$$

where

52 $${E}_{\mathrm{4,0}}\left({\epsilon }_{4},{\epsilon }_{3}\right)={\epsilon }_{4}\frac{\sqrt{{\gamma }_{3}^{2}+4{\gamma }_{2}{\gamma }_{4}+4{\epsilon }_{3}\sqrt{{\gamma }_{2}{\gamma }_{4}}}}{4\alpha },$$

53 $${E}_{\mathrm{4,1}}\left({\epsilon }_{1},{\epsilon }_{2}\right)=\frac{1}{2\alpha }\left(-\sqrt{{\gamma }_{2}{\gamma }_{4}}{\epsilon }_{1}+{\gamma }_{3}{\epsilon }_{2}\right),$$

54 $${E}_{\mathrm{4,2}}\left({\epsilon }_{4},{\epsilon }_{3},{\epsilon }_{2}\right)=-{E}_{\mathrm{4,0}}\left({\epsilon }_{4},{\epsilon }_{3}\right)-{\epsilon }_{2}\frac{\sqrt{{\gamma }_{2}{\gamma }_{4}}}{\alpha },$$

55 $${E}_{\mathrm{4,3}}\left({\epsilon }_{1}\right)={\epsilon }_{1}\frac{\sqrt{{\gamma }_{2}{\gamma }_{4}}}{2\alpha },$$

56 $${E}_{\mathrm{4,4}}\left({\epsilon }_{4},{\epsilon }_{3},{\epsilon }_{1},{\epsilon }_{2}\right)={\epsilon }_{4}\frac{{\gamma }_{2}{\gamma }_{3}{\gamma }_{4} \left|{\gamma }_{3}+2\sqrt{{\gamma }_{2}{\gamma }_{4}}\right| }{2\alpha \left({\gamma }_{3}^{2}-4{\gamma }_{2}{\gamma }_{4}\right)}-{\epsilon }_{3}\frac{\left|{\gamma }_{3}+2\sqrt{{\gamma }_{2}{\gamma }_{4}}\right|{\left({\gamma }_{2}{\gamma }_{4}\right)}^{1.5}}{\alpha \left({\gamma }_{3}^{2}-4{\gamma }_{2}{\gamma }_{4}\right)} -{\epsilon }_{1}\frac{{\gamma }_{2}{\gamma }_{4}^{2}}{2\alpha }+{\epsilon }_{2}\frac{{\gamma }_{3}\sqrt{{\gamma }_{2}{\gamma }_{4}}}{2\alpha },$$

and

57 $${E}_{\mathrm{4,5}}\left({\epsilon }_{12}\right)=-{\epsilon }_{2}\frac{{\gamma }_{2}{\gamma }_{4}}{\alpha }.$$

The solution ${\mathcal{V}}_{4.3}\left(x,t\right)$ is a singular mixed-type soliton solution that combines kink-like transitions with rational hyperbolic structures, resulting in a complex voltage waveform with both sharp edges and gradually decaying components. This solution captures the behavior of electrical signals that experience a steep transition (like a voltage step) followed by nonlinear damping or tailing effects. Such waveforms are highly relevant in modeling multi-stage switching, or waveform shaping in nonlinear lossy transmission lines, where precision in controlling both the sharpness and decay of a signal is critical for maintaining signal integrity and minimizing distortion in high-speed or sensitive electronic systems.

**Set of Solutions 5**

$$Set 5=\left\{\begin{array}{c}{\varrho }_{0}=\frac{1}{2\alpha }\left(1+\frac{{\gamma }_{2}}{\sqrt{{\gamma }_{2}^{2}-3{\gamma }_{0}{\gamma }_{4}}}\right),{\varrho }_{-2}=\frac{3{\gamma }_{0}}{2\alpha \sqrt{{\gamma }_{2}^{2}-3{\gamma }_{0}{\gamma }_{4}}},{\varrho }_{1}={\varrho }_{2}={\varrho }_{-1}={\theta }_{0}={\theta }_{1}={\theta }_{2}={b}_{3}=0,\\ {b}_{4}=\frac{{b}_{1}}{4\sqrt{{\gamma }_{2}^{2}-3{\gamma }_{0}{\gamma }_{4}}}\end{array}\right\}$$

At ${\gamma }_{1}={\gamma }_{3}=0,$ we get

58 $${\mathcal{V}}_{5.1}\left(x,t\right)={F}_{0\left(+\right)}+{F}_{1}{\mathrm{cosh}}^{2}\left(\sqrt{{\gamma }_{2}} \varkappa \left(x,t\right)\right)$$

and

59 $${\mathcal{V}}_{5.2}\left(x,t\right)={F}_{0\left(\pm \right)}+{F}_{1\left(\mp \right)}{\mathrm{cos}}^{2}\left(\sqrt{{\gamma }_{2}} \varkappa \left(x,t\right)\right).$$

${\mathcal{V}}_{5.1}\left(x,t\right)$ is a hyperbolic soliton solution, modeling a smooth, localized voltage pulse that maintains its shape during propagation, typical in nonlinear transmission lines with low loss and distributed capacitance. In contrast, ${\mathcal{V}}_{5.2}\left(x,t\right)$ (Eq. 59) is a trigonometric periodic wave, representing regular, symmetric voltage oscillations commonly seen in oscillators or modulated carrier signals. Together, these solutions reflect how nonlinearity and fractional-order effects influence signal shaping, stability, and periodicity in practical electronic and communication systems. The constants ${F}_{0\left(\pm \right)}$ and ${F}_{1\left(\pm \right)}$ have the forms

60 $${F}_{0\left(\pm \right)}=\frac{1}{2\alpha }\left(1\pm \frac{{\gamma }_{2}{\epsilon }_{1}}{\sqrt{{\gamma }_{2}^{2}-3{\gamma }_{0}{\gamma }_{4}}}\mp \frac{3{\gamma }_{0}{\epsilon }_{1}\left(\sqrt{{\gamma }_{4}^{2}-4{\gamma }_{2}{\gamma }_{6}}\pm {\gamma }_{4}\right)}{2{\gamma }_{2}\sqrt{{\gamma }_{2}^{2}-3{\gamma }_{0}{\gamma }_{4}}}\right)$$

and

61 $${F}_{1\left(\mp \right)}=\pm \frac{3{\gamma }_{0}\sqrt{{\gamma }_{4}^{2}-4{\gamma }_{2}{\gamma }_{6}}}{2\alpha {\gamma }_{2}\sqrt{{\gamma }_{2}^{2}-3{\gamma }_{0}{\gamma }_{4}}}.$$

**Sets of Solutions 6**

$$Set 6-1=\left\{{\varrho }_{2}=\frac{6{b}_{4}{\gamma }_{4}}{\alpha {b}_{1}}, {\varrho }_{0}=\pm \frac{1}{\alpha },{\varrho }_{1}={\varrho }_{-1}={\varrho }_{-2}={\theta }_{0}={\theta }_{1}={b}_{3}=0\right\}$$

$$Set 6-2=\left\{\begin{array}{c}{\varrho }_{0}=\frac{1}{2\alpha },{\varrho }_{2}=\frac{6{b}_{4}{\gamma }_{4}}{\alpha {b}_{1}}, {\theta }_{1}=\pm \frac{1}{2\alpha \sqrt{{\gamma }_{2}}},{b}_{3}=\pm \frac{5{b}_{1}}{12\sqrt{{\gamma }_{2}}},\\ {\varrho }_{1}={\varrho }_{-1}={\varrho }_{-2}={\theta }_{0}={\theta }_{2}=0\end{array}\right\}$$

$$Set 6-3=\left\{\begin{array}{c}{\varrho }_{0}=\frac{{\epsilon }_{5}}{\alpha },{\varrho }_{2}=\frac{3{\epsilon }_{6}{\gamma }_{4}}{\alpha {\gamma }_{2}},{\theta }_{0}=\frac{3{\epsilon }_{7}\sqrt{{\gamma }_{4}}}{\alpha {\gamma }_{2}},{b}_{1}={\epsilon }_{6}{b}_{4}{\gamma }_{2},\\ {\varrho }_{1}={\varrho }_{-1}={\varrho }_{-2}={\theta }_{1}={\theta }_{2}={b}_{3}=0\end{array}\right\}$$

$$Set 6-4=\left\{\begin{array}{c}{\varrho }_{0}=\frac{1}{2\alpha },{\varrho }_{1}=\frac{{\epsilon }_{8}\sqrt{{\gamma }_{4}}}{2\alpha \sqrt{{\gamma }_{2}}},{\varrho }_{2}=\frac{{\gamma }_{4}}{2\alpha {\gamma }_{2}},{\theta }_{0}=\frac{{\epsilon }_{9}\sqrt{{\gamma }_{4}}}{2\alpha {\gamma }_{2}},{\theta }_{1}=\frac{{\epsilon }_{10}}{2\alpha \sqrt{{\gamma }_{2}}},{b}_{1}=6{b}_{4}{\gamma }_{2},{b}_{3}=5{\epsilon }_{10}{b}_{4}\sqrt{{\gamma }_{2}}\\ {\varrho }_{-1}={\varrho }_{-2}={\theta }_{2}=0\end{array}\right\}$$

$$Set 6-5=\left\{\begin{array}{c}{\varrho }_{0}=\frac{1}{2\alpha },{\varrho }_{1}=\frac{{\epsilon }_{12}\sqrt{{\gamma }_{4}}}{2\alpha \sqrt{{\gamma }_{2}}},{\varrho }_{2}=-\frac{{\gamma }_{4}}{2\alpha {\gamma }_{2}},{\theta }_{0}={\epsilon }_{11}\frac{\sqrt{{\gamma }_{4}}}{2\alpha {\gamma }_{2}},\\ {\theta }_{1}=\frac{{\epsilon }_{11}}{2\alpha \sqrt{{\gamma }_{2}}},{b}_{1}=-6{b}_{4}{\gamma }_{2},{b}_{3}=-{\epsilon }_{11}5{b}_{4}\sqrt{{\gamma }_{2}} \\ , {\varrho }_{-1}={\varrho }_{-2}={\theta }_{2}=0\end{array}\right\}$$

At ${\gamma }_{1}={\gamma }_{3}={\gamma }_{6}=0$, we get different sets of solution based on Jacobi elliptic wave solutions. The corresponding set of solutions.

At $${\gamma }_{0}=1, {\gamma }_{2}=-1-{\mu }^{2}, {\gamma }_{4}={\mu }^{2}$$

62 $${\mathcal{V}}_{6.1-1}\left(x,t\right)=\pm \frac{1}{\alpha }+{G}_{1} {\mu }^{2}{sn}^{2}\left(\varkappa \left(x,t\right),\mu \right)$$

and

63 $${\mathcal{V}}_{6.1-2}\left(x,t\right)=\frac{{\epsilon }_{5}}{\alpha }+\mu {\upepsilon }_{6} {G}_{2}\left(\mu \right) {sn}^{2}\left(\varkappa \left(x,t\right),\mu \right)+{\upepsilon }_{7} {G}_{2}\left(\mu \right) cn\left(\varkappa \left(x,t\right),\mu \right) dn\left(\varkappa \left(x,t\right),\mu \right).$$

where ${G}_{1}=\frac{6 {b}_{4}}{\alpha {b}_{1}}, {G}_{2}\left(\mu \right)=-\frac{3\mu }{\alpha \left({\mu }^{2}+1\right)}$ and the set of ${\epsilon }_{j}{\prime}$ s in this equation have been defined in Table 2. The solution ${\mathcal{V}}_{6.1-1}\left(x,t\right)$ (Eq. 62) is a periodic soliton solution producing a bounded, oscillatory voltage waveform influenced by the elliptic modulus $\mu$. This is ideal for modeling nonlinear transmission lines with controlled periodic energy localization. In contrast, the solution ${\mathcal{V}}_{6.1-2}\left(x,t\right)$ (Eq. 63) extends this behavior leading to a more complex, modulated waveform with richer dynamics. This allows the modeling of hybrid periodic signals where both amplitude modulation and pulse shaping are essential in signal processing and transmission systems.

**Table 2:** The parameter arrays $\left({\epsilon }_{5}, {\epsilon }_{6}, {\epsilon }_{7}\right)$ correspond to the four different forms of the solution, where ${\epsilon }_{j}\in \left\{\mathrm{0,1},-1\right\}$.

${\epsilon }_{5}$	${\epsilon }_{6}$	${\epsilon }_{7}$
$0$	$-1$	$-1$
$0$	$-1$	$1$
$1$	$1$	$-1$
$1$	$1$	$1$

At ${\gamma }_{0}={\mu }^{2}-1, {\gamma }_{2}=2-{\mu }^{2}, {\gamma }_{4}=-1$, we get

64 $${\mathcal{V}}_{6.2-1}\left(x,t\right)=\pm \frac{1}{\alpha }+{G}_{1} {dn}^{2}\left(\varkappa \left(x,t\right),\mu \right)$$

and

65 $${\mathcal{V}}_{6.2-2}\left(x,t\right)=\frac{1}{2\alpha }-{G}_{1} {dn}^{2}\left(\varkappa \left(x,t\right),\mu \right)+{G}_{3}\left(\mu \right)\frac{cn\left(\varkappa \left(x,t\right),\mu \right) sn\left(\varkappa \left(x,t\right),\mu \right)}{dn\left(\varkappa \left(x,t\right),\mu \right)}$$

where ${G}_{3}\left(\mu \right)=-\frac{\mu }{2 \alpha \sqrt{2-{\mu }^{2}}}$. The solution ${\mathcal{V}}_{6.2-1}\left(x,t\right)$ (Eq. 64) describes a periodic soliton wave based on the squared Jacobi elliptic function. This solution produces a smooth voltage profile that retains its form over distance, making it ideal for modeling stable wave propagation in nonlinear circuits. On the other hand, ${\mathcal{V}}_{6.2-2}\left(x,t\right)$ (Eq. 65) presents a more intricate waveform resulting in a modulated periodic pulse with asymmetric features. This form is suitable for capturing nonuniform energy distributions or engineered asymmetries in advanced transmission lines or signal shaping applications.

At ${\gamma }_{0}=-{\mu }^{2}, {\gamma }_{2}=-1+2{\mu }^{2}, {\gamma }_{4}=1-{\mu }^{2}$, we get

66 $${\mathcal{V}}_{6.3-1}\left(x,t\right)=\pm \frac{1}{\alpha }+{G}_{1}\left(1-{\mu }^{2}\right) {nc}^{2}\left(\varkappa \left(x,t\right),\mu \right),$$

67 $${\mathcal{V}}_{6.3-2}\left(x,t\right)=\frac{1}{2\alpha }+{G}_{1}\left(1-{\mu }^{2}\right) {nc}^{2}\left(\varkappa \left(x,t\right),\mu \right)\pm {G}_{4}\left(\mu \right) \frac{dc\left(\varkappa \left(x,t\right),\mu \right) sc\left(\varkappa \left(x,t\right),\mu \right)}{nc\left(\varkappa \left(x,t\right),\mu \right)},$$

and

$${\mathcal{V}}_{6.3-3}\left(x,t\right)=\frac{1}{2\alpha }+{\epsilon }_{8} {G}_{5}\left(\mu \right) nc\left(\varkappa \left(x,t\right),\mu \right)+{G}_{6}\left(\mu \right) {nc}^{2}\left(\varkappa \left(x,t\right),\mu \right)$$

68 $$+{\epsilon }_{9} {G}_{4}\left(\mu \right) dc\left(\varkappa \left(x,t\right),\mu \right) sc\left(\varkappa \left(x,t\right),\mu \right)+{\epsilon }_{10} {G}_{5}\left(\mu \right)\frac{dc\left(\varkappa \left(x,t\right),\mu \right) sc\left(\varkappa \left(x,t\right),\mu \right)}{nc\left(\xi ,\mu \right)}.$$

where ${G}_{4}\left(\mu \right)=\frac{1}{2 \alpha \sqrt{2{\mu }^{2}-1}}, {G}_{5}\left(\mu \right)=\sqrt{1-{\mu }^{2}} {G}_{4}$, ${G}_{6}\left(\mu \right)=\frac{{\mu }^{2}-1}{2\alpha \left(2{\mu }^{2}-1\right)}$. Also, ${\epsilon }_{8}, {\epsilon }_{9}$ and ${\epsilon }_{10}$ are defined in Table 3.

**Table 3:** The parameter arrays $\left({\epsilon }_{8}, {\epsilon }_{9}, {\epsilon }_{10}\right)$ correspond to the four different forms of the solution, where ${\epsilon }_{j}\in \left\{1,-1\right\}$.

${\epsilon }_{8}$	${\epsilon }_{9}$	${\epsilon }_{10}$
$-1$	$-1$	$-1$
$1$	$1$	$-1$
$1$	$-1$	$1$
$-1$	$1$	$1$

The solutions ${\mathcal{V}}_{6.3-1}\left(x,t\right), {\mathcal{V}}_{6.3-2}\left(x,t\right)$ and ${\mathcal{V}}_{6.3-3}\left(x,t\right)$ (Eqs. 66 – 68) describe complex periodic waveforms using Jacobi elliptic functions. Equation (66) yields a bright-type soliton with amplitude modulation shaped by the elliptic modulus $\mu$, modeling high-frequency localized voltage spikes. Equation (67) introduces an added rational interaction resulting in asymmetric pulse structures suitable for characterizing nonlinear filtering or asymmetric waveguiding. Equation (68) presents the most intricate profile, combining linear and rational interactions among elliptic functions to capture hybrid wave behaviors with controllable peaks and valleys. These solutions offer valuable models for engineering nonlinear electrical media, enabling precise control over pulse shape, amplitude, and periodicity in real-world signal processing and transmission systems.

In addition, the set of solutions has been obtained,

69 $${\mathcal{V}}_{6.3-4}\left(x,t\right)=\frac{{\epsilon }_{5}}{\alpha }-6 {\epsilon }_{6} {G}_{6}\left(\mu \right) {nc}^{2}\left(\varkappa \left(x,t\right),\mu \right) +6 {\epsilon }_{7} {G}_{7}\left(\mu \right) dc\left(\varkappa \left(x,t\right),\mu \right) sc\left(\varkappa \left(x,t\right),\mu \right)$$

and

70 $${\mathcal{V}}_{6.3-5}\left(x,t\right)=\frac{1}{2\alpha }+{G}_{8}\left(\mu \right) {nc}^{2}\left(\varkappa \left(x,t\right),\mu \right)+{G}_{7}\left(\mu \right) dc\left(\varkappa \left(x,t\right),\mu \right) sc\left(\varkappa \left(x,t\right),\mu \right) \pm {G}_{4}\left(\mu \right)\frac{dc\left(\varkappa \left(x,t\right),\mu \right) sc\left(\varkappa \left(x,t\right),\mu \right)}{ nc\left(\varkappa \left(x,t\right),\mu \right)}$$

where $${G}_{7}\left(\mu \right)=\frac{\sqrt{1-{\mu }^{2}}}{2 \alpha \left(2{\mu }^{2}-1\right)}$$ and $${G}_{8}\left(\mu \right)=-\frac{{\mu }^{4}}{4\alpha \left({\mu }^{2}-2\right)}.$$

The solutions ${\mathcal{V}}_{6.3-4}\left(x,t\right)$ and ${\mathcal{V}}_{6.3-5}\left(x,t\right)$ (Eqs. 69 – 70) expand upon the Jacobi elliptic-based waveforms within the reciprocal elliptic function domain, modeling nonlinear wave dynamics with refined structure. This setup enables a tunable control over polarity and wave symmetry, making it suitable for systems requiring abrupt switching or bistable responses. Equation (70) introduces a layered configuration combining symmetric and asymmetric components, allowing for more flexible waveform shaping. These expressions are particularly relevant for describing elliptic solitons and engineered periodic pulses in nonlinear lattices or transmission media where fine control over waveform features is critical.

At ${\gamma }_{0}=-1, {\gamma }_{2}=2-{\mu }^{2}, {\gamma }_{4}={\mu }^{2}-1$, we get

71 $${\mathcal{V}}_{6.4-1}\left(x,t\right)=\pm \frac{1}{\alpha }+{G}_{1}\left({\mu }^{2}-1\right) {nd}^{2}\left(\varkappa \left(x,t\right),\mu \right),$$

72 $${\mathcal{V}}_{6.4-2}\left(x,t\right)=\frac{1}{2\alpha }+{G}_{1} \left({\mu }^{2}-1\right) {nd}^{2}\left(\varkappa \left(x,t\right),\mu \right)\pm {G}_{3}\left(\mu \right)\frac{cd\left(\varkappa \left(x,t\right),\mu \right) sd\left(\varkappa \left(x,t\right),\mu \right)}{nd\left(\varkappa \left(x,t\right),\mu \right)},$$

$${\mathcal{V}}_{6.4-3}\left(x,t\right)=-{\epsilon }_{12} {G}_{3}\left(\mu \right) \left[\frac{\sqrt{2}}{\mu }\frac{dn\left(\varkappa \left(x,t\right),\mu \right)cn\left(\varkappa \left(x,t\right),\mu \right)}{sn\left(\varkappa \left(x,t\right),\mu \right)}+\frac{ sn\left(\varkappa \left(x,t\right),\mu \right)}{\left(1+dn\left(\varkappa \left(x,t\right),\mu \right)\right)}\left(\frac{\mu }{\sqrt{2}}+cn\left(\varkappa \left(x,t\right),\mu \right)\right)\right]+{G}_{8}\left(\mu \right)\frac{{sn}^{2}\left(\varkappa \left(x,t\right),\mu \right)}{{\left(1+dn\left(\varkappa \left(x,t\right),\mu \right)\right)}^{2}}$$

73 $$-\frac{2{\epsilon }_{11}}{{\mu }^{2}}{G}_{8}\left(\mu \right)\frac{cn\left(\varkappa \left(x,t\right),\mu \right)dn\left(\varkappa \left(x,t\right),\mu \right)}{\left(1+dn\left(\varkappa \left(x,t\right),\mu \right)\right)}-\frac{2{\epsilon }_{11}}{\mu }{G}_{8}\left(\mu \right)\frac{cn\left(\varkappa \left(x,t\right),\mu \right) {sn}^{2}\left(\varkappa \left(x,t\right),\mu \right) }{{\left(1+dn\left(\varkappa \left(x,t\right),\mu \right)\right)}^{2}}$$

and

74 $${\mathcal{V}}_{6.4-4}\left(x,t\right)=\frac{{\epsilon }_{5}}{\alpha }+6 {\epsilon }_{6} {G}_{8}\left(\mu \right)\frac{{sn}^{2}\left(\varkappa \left(x,t\right),\mu \right)}{{\left(1+dn\left(\varkappa \left(x,t\right),\mu \right)\right)}^{2}}+{\epsilon }_{7}\frac{12}{{\mu }^{2}}{G}_{8}\left(\mu \right)\frac{cn\left(\varkappa \left(x,t\right),\mu \right) }{1+dn\left(\varkappa \left(x,t\right),\mu \right)}\left(dn\left(\varkappa \left(x,t\right),\mu \right)+\frac{\mu {sn}^{2}\left(\varkappa \left(x,t\right),\mu \right)}{1+dn\left(\varkappa \left(x,t\right),\mu \right)}\right).$$

In addition, we get

75 $$\begin{gathered} {\mathcal{V}}_{{6.4 - 5}} \left( {x,t} \right) = \frac{1}{{2\alpha }} + \frac{{G_{8} \left( \mu \right)}}{{1 + dn\left( {\xi ,\mu } \right)}} \hfill \\ \left[ { - \frac{{sn^{2} \left( {\left( {x,t} \right),\mu } \right)}}{{1 + dn\left( {\left( {x,t} \right),\mu } \right)}} + \in _{{11}} \frac{2}{{\mu ^{2} }}cn\left( {\left( {x,t} \right),\mu } \right)~dn\left( {\left( {x,t} \right),\mu } \right)} \right. \hfill \\ \left. { + \in _{{11}} ~\mu ^{2} \frac{{cn\left( {\left( {x,t} \right),\mu } \right)~sn^{2} \left( {\left( {x,t} \right),\mu } \right)}}{{1 + dn\left( {\xi ,\mu } \right)}}} \right] \hfill \\ + \frac{{G_{3} \left( \mu \right)}}{{1 + dn\left( {\left( {x,t} \right),\mu } \right)}}\left[ { \in _{{11}} \frac{\mu }{{\sqrt 2 }}{\mathrm{sn}}\left( {\left( {x,t} \right),\mu } \right) + \frac{{ \in _{{12}} }}{{\sqrt 2 \mu }}\frac{{cn\left( {\left( {x,t} \right),\mu } \right)}}{{{\mathrm{sn}}\left( {\left( {x,t} \right),\mu } \right)}}} \right. \hfill \\ \left. {\left( {dn\left( {\xi ,\mu } \right) + dn^{2} \left( {\left( {x,t} \right),\mu } \right)} \right) + \in _{{12}} \frac{{cn\left( {\left( {x,t} \right),\mu } \right){\mathrm{~}}sn\left( {\left( {x,t} \right),\mu } \right)}}{{\left( {1 + dn\left( {\left( {x,t} \right),\mu } \right)} \right)^{2} }}\left( { - \frac{1}{\mu } + \sqrt 2 ~dn\left( {\left( {x,t} \right),\mu } \right)} \right)} \right] \hfill \\ \end{gathered} .$$

The solutions from ${\mathcal{V}}_{6.4-1}\left(x,t\right)$ to ${\mathcal{V}}_{6.4-5}\left(x,t\right)$ (Eqs. 71–75) represent a diverse set of elliptic soliton forms involving reciprocal Jacobi elliptic functions, capturing a wide range of nonlinear wave behaviors in periodic media. Equation (71) describes a symmetric solution which is suitable for modeling bistable or symmetric transmission. Equation (72) adds an asymmetric perturbation that introduces directional behavior or signal asymmetry. The more intricate structure in Eq. (73) is controlled by binary parameters $\left({\epsilon }_{11},{\epsilon }_{12}\right)$ that encode different waveform families (Table 4), representing higher-order interactions and waveform transformations. Equation (74) generalizes these forms by introducing new ternary parameter arrays $( \in _{{13}} , \in _{{14}} ,~ \in _{{15}} )$ to switch between waveform amplitudes and polarities (Table 5). This enables adaptive modulation effects. Lastly, the complex form in (75) incorporates coupled rational terms enabling precise engineering of nonlinear oscillations, phase shifts, or envelopes shaping in transmission lines and photonic lattices. Together, these solutions enrich the catalog of analytic solitons in nonlinear electrical systems and provide exact waveform templates for tuning signal propagation in engineered media.

**Table 4:** The parameter arrays $\left({\epsilon }_{11}, {\epsilon }_{12}\right)$ correspond to the four different forms of the solution, where ${\epsilon }_{j}\in \left\{1,-1\right\}$.

${\epsilon }_{11}$	${\epsilon }_{12}$
$-1$	$-1$
$1$	$-1$
$-1$	$1$
$1$	$1$

**Table 5:** The parameter arrays $\left({\epsilon }_{13}, {\epsilon }_{14},{\epsilon }_{15}\right)$ correspond to the four different forms of the solution, where ${\epsilon }_{j}\in \left\{\mathrm{0,1},-1\right\}$.

${\epsilon }_{13}$	${\epsilon }_{14}$	${\epsilon }_{15}$
$0$	$-1$	$-1$
$0$	$-1$	$1$
$1$	$1$	$-1$
$1$	$1$	$1$

At ${\gamma }_{0}=-2{\mu }^{3}+{\mu }^{4}+{\mu }^{2}, {\gamma }_{2}=-4/\mu , {\gamma }_{4}=6\mu -{\mu }^{2}-1$, we get

76 $${\mathcal{V}}_{6.5-1}\left(x,t\right)=\pm \frac{1}{\alpha }+{G}_{9}\left(\mu \right)\frac{{cn}^{2}\left(\varkappa \left(x,t\right),\mu \right) {dn}^{2}\left(\varkappa \left(x,t\right),\mu \right)}{{(1+\mu {sn}^{2}\left(\varkappa \left(x,t\right),\mu \right))}^{2}}$$

where $${G}_{9}\left(\mu \right)=\frac{6{\mu }^{2}(-1+6\mu -{\mu }^{2}){b}_{4}}{\alpha {b}_{1}}$$

77 $$\begin{gathered} {\mathcal{V}}_{{6.5 - 2}} \left( {x,t} \right) = \frac{{ \in _{{13}} }}{\alpha } - \in _{{14}} \frac{{3\mu ^{3} \left( { - 1 + 6\mu - \mu ^{2} } \right)}}{{4\alpha }}\frac{{cn^{2} \left( {\left( {x,t} \right),\mu } \right)dn^{2} \left( {\left( {x,t} \right),\mu } \right)}}{{\left( {1 + \mu ~sn^{2} \left( {\left( {x,t} \right),\mu } \right)} \right)^{2} }} \hfill \\ ~ + \in _{{15}} \frac{{3\mu \sqrt { - 1 + 6\mu - \mu ^{2} } }}{{4\alpha }}\frac{\mu }{{1 + \mu ~sn^{2} \left( {\left( {x,t} \right),\mu } \right)}} \hfill \\ \left[ {\frac{{2\mu cn^{2} \left( {\left( {x,t} \right),\mu } \right)dn^{2} \left( {\left( {x,t} \right),\mu } \right)sn\left( {\left( {x,t} \right),\mu } \right)}}{{1 + \mu ~sn^{2} \left( {\left( {x,t} \right),\mu } \right)}}} \right. \hfill \\ + \left. {\mu ~cn^{2} \left( {\left( {x,t} \right),\mu } \right)sn\left( {\left( {x,t} \right),\mu } \right) + dn^{2} \left( {\left( {x,t} \right),\mu } \right)~sn^{2} \left( {\left( {x,t} \right),\mu } \right)} \right] \hfill \\ \end{gathered}$$

The solutions ${\mathcal{V}}_{6.5-1}\left(x,t\right)$ and ${\mathcal{V}}_{6.5-2}\left(x,t\right)$, given in Eqs. (76) and (77), describe elliptic solitons involving Jacobi functions. Both solutions capture modulated, localized waveforms shaped by the elliptic parameter $\mu$.

At ${\gamma }_{0}=1/4, {\gamma }_{2}=0.5{\mu }^{2}-1, {\gamma }_{4}={\mu }^{4}/4$, the following set of solutions has been obtained:

78 $${\mathcal{V}}_{6.6-1}\left(x,t\right)=\pm \frac{1}{\alpha }+\frac{{\mu }^{4}}{2}{G}_{1}\frac{{sn}^{2}\left(\varkappa \left(x,t\right),\mu \right)}{{\left(1+dn\left(\varkappa \left(x,t\right),\mu \right)\right)}^{2}}$$

and

79 $${\mathcal{V}}_{6.6-2}\left(x,t\right)=\frac{1}{2\alpha }\pm \mu { G}_{15}\left(\mu \right)\frac{cn\left(\varkappa \left(x,t\right),\mu \right) sn\left(\varkappa \left(x,t\right),\mu \right)}{1+dn\left(\varkappa \left(x,t\right),\mu \right)}$$

where ${G}_{15}\left(\mu \right)=\frac{1}{\alpha \sqrt{2{\mu }^{2}-4}}$*.* Additionally, ${\mathcal{V}}_{6.6-3}\left(x,t\right), {\mathcal{V}}_{6.6-4}\left(x,t\right)$ *and* ${\mathcal{V}}_{6.6-5}\left(x,t\right)$ have been found

80 $$\begin{gathered} {\mathcal{V}}_{6.6 - 3} \left( {x,t} \right) = \frac{1}{2\alpha } + \in_{8} \frac{{\mu^{2} }}{2} G_{15} \left( \mu \right)\frac{{{ }sn\left( {\left( {x,t} \right),\mu } \right)}}{{\left( {1 + dn\left( {\left( {x,t} \right),\mu } \right)} \right)}} \hfill \\ + \in_{9} G_{16} \left( \mu \right)\frac{{cn\left( {\left( {x,t} \right),\mu } \right) dn\left( {\left( {x,t} \right),\mu } \right)}}{{1 + dn\left( {\left( {x,t} \right),\mu } \right)}} + \in_{10} G_{15} \left( \mu \right)\frac{{cn\left( {\left( {x,t} \right),\mu } \right) dn\left( {\left( {x,t} \right),\mu } \right)}}{{\left( {1 + dn\left( {\left( {x,t} \right),\mu } \right)} \right){ }sn\left( {\left( {x,t} \right),\mu } \right)}} \hfill \\ + \in_{10} G_{15} \left( \mu \right)\frac{{cn\left( {\left( {x,t} \right),\mu } \right) dn^{2} \left( {\left( {x,t} \right),\mu } \right)}}{{(\left( {1 + dn\left( {\left( {x,t} \right),\mu } \right)} \right){ }sn\left( {\left( {x,t} \right),\mu } \right)}} - \frac{{\mu^{2} }}{2} G_{16} \left( \mu \right)\frac{{sn^{2} \left( {\left( {x,t} \right),\mu } \right)}}{{\left( {1 + dn\left( {\left( {x,t} \right),\mu } \right)} \right)^{2} }} \hfill \\ \in_{9} \mu G_{16} \left( \mu \right)\frac{{cn\left( {\left( {x,t} \right),\mu } \right) sn^{2} \left( {\left( {x,t} \right),\mu } \right)}}{{\left( {1 + dn\left( {\left( {x,t} \right),\mu } \right)} \right)^{2} }} + \in_{10} \mu G_{15} \left( \mu \right)\frac{{cn\left( {\left( {x,t} \right),\mu } \right){ }sn\left( {\left( {x,t} \right),\mu } \right)}}{{\left( {1 + dn\left( {\left( {x,t} \right),\mu } \right)} \right)^{2} }} \hfill \\ + \in_{10} \mu G_{15} \left( \mu \right)\frac{{cn\left( {\left( {x,t} \right),\mu } \right) dn\left( {\left( {x,t} \right),\mu } \right){ }sn\left( {\left( {x,t} \right),\mu } \right)}}{{\left( {1 + dn\left( {\left( {x,t} \right),\mu } \right)} \right)^{2} }}, \hfill \\ \end{gathered}$$

where $${G}_{16}\left(\mu \right)=\frac{{\mu }^{2}}{2\alpha ({\mu }^{2}-2)}$$

81 $$\begin{gathered} {\mathcal{V}}_{6.6 - 4} \left( {x,t} \right) = \frac{{ \in_{5} }}{\alpha }\frac{1}{{\left( {1 + dn\left( {\left( {x,t} \right),\mu } \right)} \right)^{2} }} + \frac{{2 \in_{5} }}{\alpha }\frac{{dn\left( {\left( {x,t} \right),\mu } \right)}}{{\left( {1 + dn\left( {\left( {x,t} \right),\mu } \right)} \right)^{2} }} \hfill \\ + \frac{{ \in_{5} }}{\alpha }\frac{{dn^{2} \left( {\left( {x,t} \right),\mu } \right)}}{{\left( {1 + dn\left( {\left( {x,t} \right),\mu } \right)} \right)^{2} }} + 3\mu^{2} \in_{6} G_{16} \left( \mu \right)\frac{{sn^{2} \left( {\left( {x,t} \right),\mu } \right)}}{{\left( {1 + dn\left( {\left( {x,t} \right),\mu } \right)} \right)^{2} }} \hfill \\ + 6 \in_{6} G_{16} \left( \mu \right)\frac{{cn\left( {\left( {x,t} \right),\mu } \right) dn\left( {\left( {x,t} \right),\mu } \right)}}{{\left( {1 + dn\left( {\left( {x,t} \right),\mu } \right)} \right)^{2} }} + 6 \in_{7} G_{16} \left( \mu \right)\frac{{cn\left( {\left( {x,t} \right),\mu } \right) dn^{2} \left( {\left( {x,t} \right),\mu } \right)}}{{\left( {1 + dn\left( {\left( {x,t} \right),\mu } \right)} \right)^{2} }} \hfill \\ + 6 \mu \in_{7} G_{16} \left( \mu \right)\frac{{cn\left( {\left( {x,t} \right),\mu } \right) sn^{2} \left( {\left( {x,t} \right),\mu } \right)}}{{\left( {1 + dn\left( {\left( {x,t} \right),\mu } \right)} \right)^{2} }}, \hfill \\ \end{gathered}$$

82 $$\begin{gathered} {\mathcal{V}}_{6.6 - 5} \left( {x,t} \right) = \frac{1}{2\alpha } + \frac{{\mu^{2} \in_{11} }}{2} G_{15} \left( \mu \right)\frac{{sn\left( {\left( {x,t} \right),\mu } \right)}}{{\left( {1 + dn\left( {\left( {x,t} \right),\mu } \right)} \right)}} \hfill \\ + \in_{11} G_{16} \left( \mu \right)\frac{{cn\left( {\left( {x,t} \right),\mu } \right) dn\left( {\left( {x,t} \right),\mu } \right)}}{{\left( {1 + dn\left( {\left( {x,t} \right),\mu } \right)} \right)}} + \in_{11} G_{16} \left( \mu \right)\frac{{cn\left( {\left( {x,t} \right),\mu } \right) dn\left( {\left( {x,t} \right),\mu } \right)}}{{\left( {1 + dn\left( {\left( {x,t} \right),\mu } \right)} \right) sn\left( {\left( {x,t} \right),\mu } \right)}} \hfill \\ + \in_{12} G_{15} \left( \mu \right)\frac{{cn\left( {\left( {x,t} \right),\mu } \right) dn^{2} \left( {\left( {x,t} \right),\mu } \right)}}{{\left( {1 + dn\left( {\left( {x,t} \right),\mu } \right)} \right) sn\left( {\left( {x,t} \right),\mu } \right)}} + \frac{{\mu^{2} }}{2}G_{11} \left( \mu \right)\frac{{sn^{2} \left( {\left( {x,t} \right),\mu } \right)}}{{\left( {1 + dn\left( {\left( {x,t} \right),\mu } \right)} \right)^{2} }} \hfill \\ + \mu \in_{11} G_{16} \left( \mu \right)\frac{{cn\left( {\left( {x,t} \right),\mu } \right) sn^{2} \left( {\left( {x,t} \right),\mu } \right)}}{{\left( {1 + dn\left( {\left( {x,t} \right),\mu } \right)} \right)^{2} }} + \mu \in_{12} G_{15} \left( \mu \right)\frac{{cn\left( {\left( {x,t} \right),\mu } \right){ }sn\left( {\left( {x,t} \right),\mu } \right)}}{{\left( {1 + dn\left( {\left( {x,t} \right),\mu } \right)} \right)^{2} }} \hfill \\ + \mu \in_{12} G_{15} \left( \mu \right)\frac{{cn\left( {\left( {x,t} \right),\mu } \right){ }dn\left( {\left( {x,t} \right),\mu } \right) sn\left( {\left( {x,t} \right),\mu } \right)}}{{\left( {1 + dn\left( {\left( {x,t} \right),\mu } \right)} \right)^{2} }}. \hfill \\ \end{gathered}$$

The solutions ${\mathcal{V}}_{6.6-1}\left(x,t\right)$ through ${\mathcal{V}}_{6.6-5}\left(x,t\right)$, given in Eqs. (78) – (82), represent a family of elliptic-type expressions derived using Jacobi elliptic functions. Equation (78) is a compact solitary wave solution, while Eq. (79) expands on this by introducing mixed $sn\left(.\right), cn\left(.\right)$, and $dn\left(.\right)$ interactions. The highly composite solution in Eq. (80) includes layered nonlinear terms weighted by parameters ${\epsilon }_{8}$ to ${\epsilon }_{10}$. Equation (81) generalizes the structure further with purely rational expressions built on squared $dn\left(.\right)$, while Eq. (82) serves as a full-scale expansion combining all previous functional patterns with finely tuned control via the ${\epsilon }_{j}{\prime}s$ parameters. These equations exhibit rich, localized, and structurally tunable soliton dynamics governed by the modulus $\mu$.

### Graphical representations of the obtained solutions

In this section novel soliton solutions have been explored for Loss-NLETL, derived using the Mod–EM method. The diverse physical structures and dynamic behaviors of these analytical solutions have been visualized through 2D, 3D, and density plots. These plots are hyperbolic, dark, singular periodic, kink-type, exponential, and Jacobi elliptic waves. Our findings provide key insights into wave propagation and stability within Loss-NLETL and many of them are new in comparison to literature. In addition, the visualizations also show how the fractional parameter changes influence soliton shape. Beyond advancing soliton theory, these results have practical implications for optimizing wave behavior in Loss-NLETL circuit design.

**Fig. 2:**
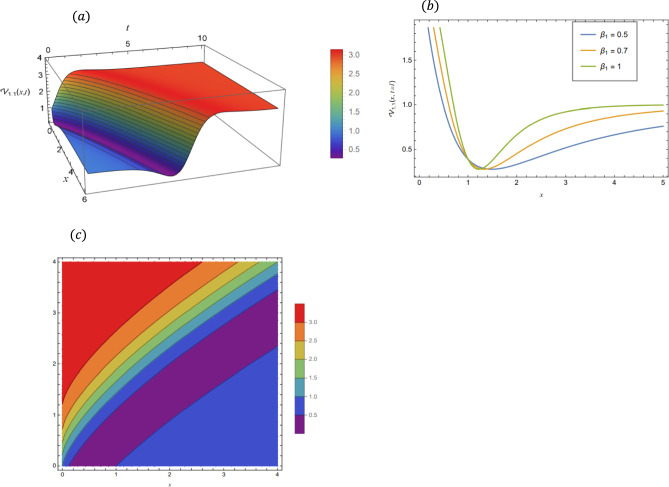
Composite hyperbolic-type solution for ${\mathcal{V}}_{1.1}(x,t)$ as given by Eq. (47) illustrating the localized structure and temporal evolution of the solution. Figure 2(a) presents a 3D surface plot over the domain $0\le x\le 6, 0\le t\le 10$. Figure 2(b) shows 2D profile plots at fixed time $t=1$ for ${\beta }_{1}=\mathrm{0.5,0.7,1}$, within the interval $0\le x\le 5$. Figure 2(c) displays a density (contour) plot over $0\le x\le 5, 0\le t\le 4$.

**Fig. 3:**
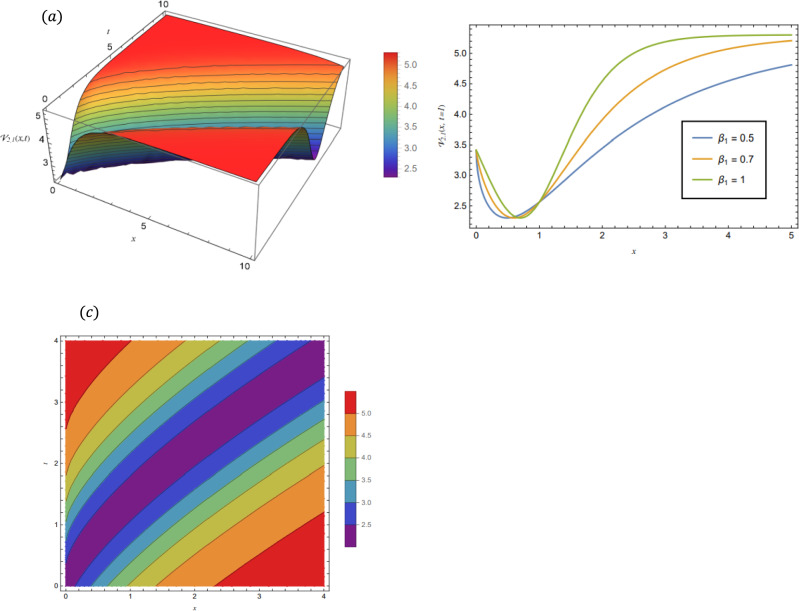
Dark soliton solution for ${\mathcal{V}}_{2.1}(x,t)$ as given by Eq. (40), illustrating the localized structure and temporal evolution of the solution. Figure 3(a) presents a 3D surface plot over the domain $0\le x\le 10$, $0\le t\le 10$. Figure 3(b) shows 2D profile plots at fixed time $t=1$ for ${\beta }_{1}=\mathrm{0.5,0.7,1}$, within the interval $0\le x\le 5$. Figure 3(c) displays a density (contour) plot over $0\le x\le 4, 0\le t\le 4$.

**Fig. 4:**
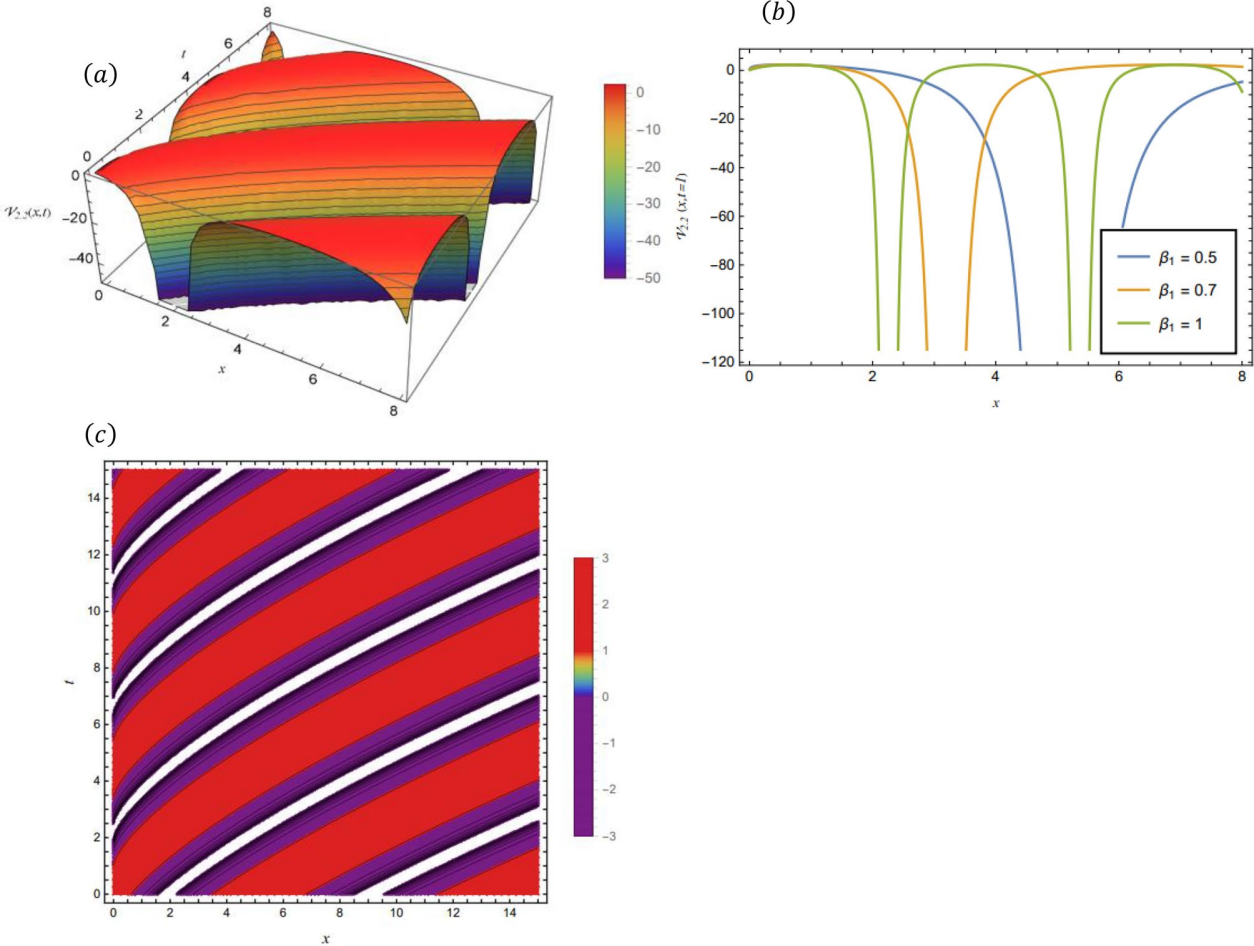
Singular periodic solution for ${\mathcal{V}}_{2.2}\left(x,t\right)$ as given by Eq. (41), illustrating the localized structure and temporal evolution of the solution. Figure 4(a) presents a 3D surface plot over the domain $0\le x\le 8$, $0\le t\le 8$. Figure 4(b) shows 2D profile plots at fixed time $t=1$ for different values of ${\beta }_{1}=0.5, 0.7, 1$, within the interval $0\le x\le 8$. Figure 4(c) displays a density (contour) plot over $0\le x\le 15, 0\le t\le 15$.

**Fig. 5:**
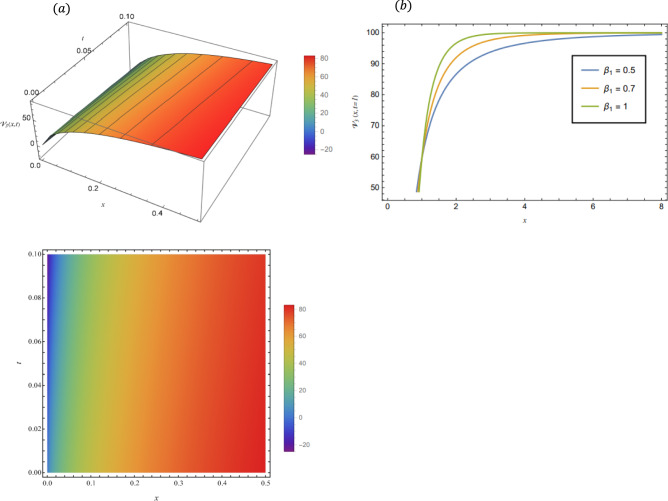
Exponential traveling wave solution for ${\mathcal{V}}_{3}\left(x,t\right)$ as given by Eq. (42), illustrating the localized structure and propagation behavior of the solution. Figure 5(a) presents a 3D surface plot over the domain $0\le x\le 0.5, 0\le t\le 0.1$. Figure 5(b) shows 2 D profile plots at fixed time $t=1$ for different values of ${\beta }_{1}=\mathrm{0.5,0.7,1}$, within the interval $0\le x\le 0.5$. Figure 5(c) displays a density (contour) plot over $0\le x\le \mathrm{0.5,0}\le t\le 0.1$.

**Fig. 6:**
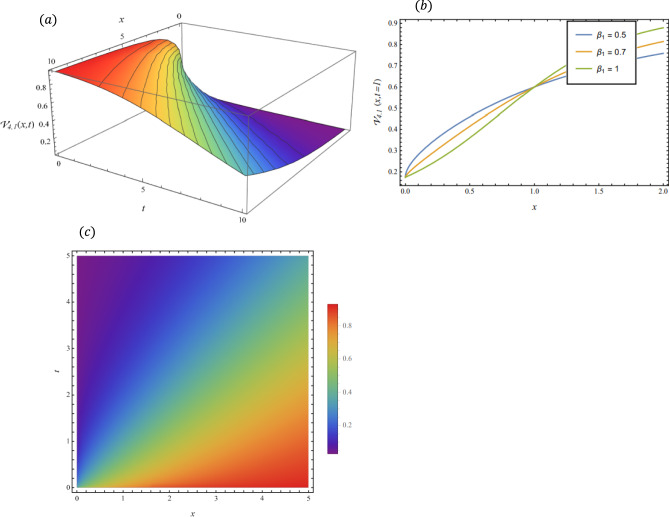
hyperbolic wave solution for ${\mathcal{V}}_{4.1}\left(x,t\right)$ as given by Eq. (43), illustrating the localized structure and propagation behavior of the solution. Figure 5(a) presents a 3D surface plot over the domain $0\le x\le 10, 0\le t\le 10$. Figure 5(b) shows 2 D profile plots at fixed time $t=1$ for different values of ${\beta }_{1}=\mathrm{0.5,0.7,1}$, within the interval $0\le x\le 2$. Figure 5(c) displays a density (contour) plot over $0\le x\le \mathrm{5,0}\le t\le 5$.

**Fig. 7:**
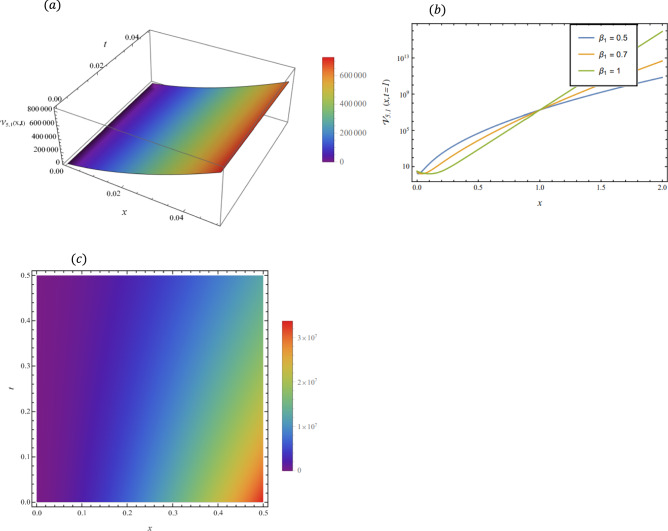
hyperbolic solution for ${\mathcal{V}}_{5.1}\left(x,t\right)$ as given by Eq. (58), illustrating the localized structure and propagation behavior of the solution. Figure 5(a) presents a 3D surface plot over the domain $0\le x\le 0.05, 0\le t\le 0.04$. Figure 5(b) shows 2 D profile plots at fixed time $t=1$ for different values of ${\beta }_{1}=\mathrm{0.5,0.7,1}$, within the interval $0\le x\le 2$. Figure 5(c) displays a density plot over $0\le x\le \mathrm{0.5,0}\le t\le 0.5$.

By adopting of the numerical values of the arbitrary parameters within appropriate ranges, several wave solutions have been plotted and are illustrated in the Figs. 2, 3, 4, 5, 6 and 7. The spatial fractional parameters ${\beta }_{1}$ is set to be $0.7$ in all the 3D and density plot displays. Regarding the temporal parameter ${\beta }_{2}$, it has been fixed to be unity. On the other hand, to show how it affects the behavior of the generated solutions, the 2D plots show the solution functions for different values of ${\beta }_{1}$. Figure 2 presents a composite hyperbolic-type solution for ${\mathcal{V}}_{1.1}(x,t)$ (Eq. (38)) using the parameters ${m}_{1}={m}_{2}=1,\alpha =1,{b}_{1}={b}_{4}=1,{\gamma }_{2}=1$ and ${\gamma }_{4}=-1$. Figure 3 displays dark soliton solution for ${\mathcal{V}}_{2.1}\left(x,t\right)$, (Eq. (40)) with the parameters ${m}_{1}={m}_{2}=1, , \alpha =1, {b}_{1}={b}_{4}=1, {\gamma }_{2}=-2.2$ and ${\gamma }_{4}=4$. A singular periodic solution is shown in Fig. 4 for ${\mathcal{V}}_{2.2}(x,t)$ (Eq. (41)) with the parameters ${m}_{1}={m}_{2}=1, \alpha =1, {b}_{1}={b}_{4}=1$ and ${\gamma }_{2}=2.2$. In addition, Fig. 5 illustrates an exponential traveling wave solution for ${\mathcal{V}}_{3}(x,t)$ (Eq. (42)) with the parameters ${m}_{1}={m}_{2}=1, \alpha =1, {b}_{1}={b}_{4}=1$ and ${\gamma }_{2}=2.2$. Figure 6 presents a periodic trigonometric wave solution for ${\mathcal{V}}_{5.2}(x,t)$ (Eq. (59)) using the parameters ${m}_{1}={m}_{2}=1, {\gamma }_{0}={\gamma }_{2}=2, {\gamma }_{4}=0.5$ and ${\gamma }_{6}=0.1$. Jacobi elliptic wave solution is displayed in Fig. 7 for ${\mathcal{V}}_{6.2-1}(x,t)$ (Eq. (64)) with the parameters ${m}_{1}={m}_{2}=1, \alpha =1, {b}_{1}={b}_{4}=1$ and $\mu =0.7$.

Figure 2 visualizes the composite hyperbolic type of solution from Eq. (38), which manifests as a kink-like step wave which is a smooth, asymmetric voltage pulse similar to a signal switch. The 3D surface and contour maps (a and c) track this distinct waveform as it propagates across space and time, while the 2D figure (b) details the pulse’s profile, demonstrating how the spatial fractional parameter ${\beta }_{1}$ sharpens this signal transition.

Figure 3 visualizes the dark soliton solution derived in Eq. (40), which appears as a distinct “dip” or valley traveling through a constant background signal. This solution represents a localized reduction in intensity. The 3D surface and contour plots (a and c) track this moving depression across space and time, while the 2D snapshot (b) reveals the specific shape of the "well," showing how adjusting the spatial fractional parameter ${\beta }_{1}$ modifies the profile of this voltage drop.

Figure 4 visualizes the singular periodic solution from Eq. (41), which manifests as a periodic traveling wave characterized by rhythmic, repeating cycles rather than a single localized pulse. This waveform is sharply interrupted by distinct “singularities” steep vertical drops in intensity where the signal breaks. The 3D surface and contour maps (a and c) track these repeating bands as they propagate across space and time, while the 2D snapshot (b) details the recurring profile, demonstrating how the spatial fractional parameter ${\beta }_{1}$ modifies the width and spacing of these periodic structures.

Figure 5 visualizes the exponential traveling wave solution from Eq. (42), which manifests as an attenuated voltage pulse-a transient signal that naturally decays or evolves due to energy dissipation, much like a pulse fading along a lossy transmission line. The 3D surface and contour maps (a and c) illustrate this propagation behavior across space and time, while the 2D snapshot (b) details the specific profile of the waveform, demonstrating how the spatial fractional parameter ${\beta }_{1}$ controls the steepness of this exponential transition.

Figure 6 visualizes the hyperbolic wave solution derived in Eq. (43), which manifests as a kink soliton-a steep voltage transition that resembles a sharp switch or nonlinear edge shaping in a high-speed circuit. The 3D surface and contour maps (a and c) track this evolving transition as it propagates across space and time, while the 2D snapshot (b) details the pulse’s rising profile, demonstrating how the fractional spatial parameter ${\beta }_{1}$ controls the steepness of this signal shift.

Figure 7 visualizes the hyperbolic solution derived in Eq. (58), which manifests as a rapidly amplifying wave. This is a signal characterized by a steep, localized surge in intensity rather than a bounded pulse. The 3D surface and density plots (a and c) map this intense energy accumulation as it evolves over space and time, while the 2D snapshot (b) captures the sharp upward trajectory of the voltage profile, highlighting how the ${\beta }_{1}$ parameter dictates the acceleration of this signal growth.

**Fig. 8:**
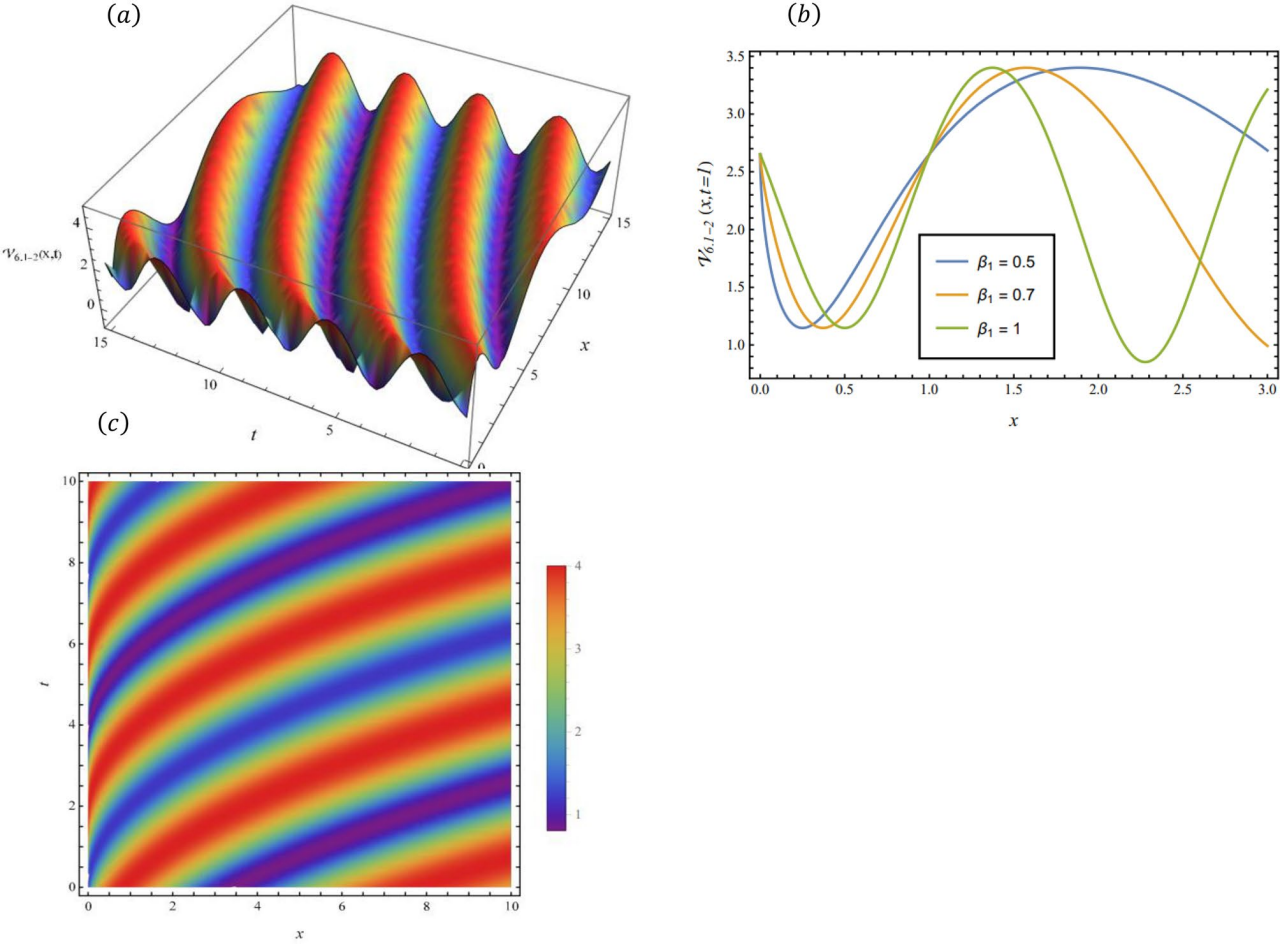
Jacobbi Elleptic wave solution for ${\mathcal{V}}_{6.1-2}\left(x,t\right)$ as given by Eq. (63), illustrating the localized structure and propagation behavior of the solution. Figure 5(a) presents a 3D surface plot over the domain $0\le x\le 15, 0\le t\le 15$. Figure 5(b) shows 2 D profile plots at fixed time $t=1$ for different values of ${\beta }_{1}=\mathrm{0.5,0.7,1}$, within the interval $0\le x\le 3$. Figure 5(c) displays a density plot over $0\le x\le 10,0\le t\le 10$.

Figure 8 visualizes the Jacobi elliptic wave solution from Eq. (63), which manifests as a complex modulated waveform. It is a sophisticated, recurring signal that combines oscillatory patterns to create a “hybrid” periodic structure often seen in amplitude modulation and pulse shaping. The 3D surface and contour maps (a and c) track these rhythmic, repeating energy bands as they propagate across space and time, while the 2D snapshot (b) reveals the intricate detail of the oscillation cycles, demonstrating how the ${\beta }_{1}$ parameter fine-tunes the shape and frequency of these periodic dynamics.

**Fig. 9:**
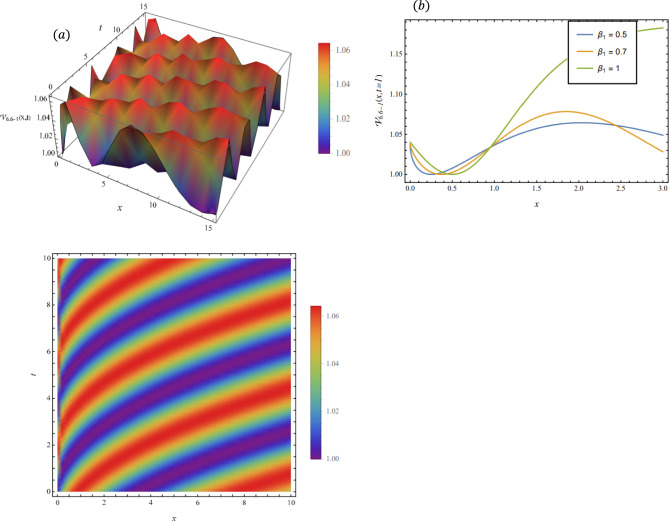
Exponential traveling wave solution for ${\mathcal{V}}_{6.6-1}\left(x,t\right)$ as given by Eq. (78), illustrating the localized structure and propagation behavior of the solution. Figure 5(a) presents a 3D surface plot over the domain $0\le x\le 15, 0\le t\le 15$. Figure 5(b) shows 2 D profile plots at fixed time $t=1$ for different values of ${\beta }_{1}=\mathrm{0.5,0.7,1}$, within the interval $0\le x\le 3$. Figure 5(c) displays a density plot over $0\le x\le 10,0\le t\le 10$.

Figure 9 visualizes the rational Jacobi elliptic solution derived in Eq. (78), which manifests as a structured periodic lattice. It is a rhythmic, recurring wave pattern that maintains a uniform, bounded shape as it travels, unlike the decaying or singular pulses seen earlier. The 3D surface and contour maps (a and c) illustrate this organized propagation, resembling a moving series of interlocked peaks and valleys, while the 2D snapshot (b) details the specific curvature of the oscillation cycles, showing how the ${\beta }_{1}$ parameter fine-tunes the amplitude and flow of this periodic signal.

In Table 6, a comparison is presented between the solutions obtained in the current work and those reported in Ref.^28^. The results demonstrate that the current study yields a broader spectrum of exact solutions, including composite, dark, hyperbolic, kink-type, and Jacobi elliptic waves, many of which are not found in Ref.^28^. By contrast, Ref.^28^ reports only a limited subset—namely singular periodic, periodic trigonometric, bright, and solitary solutions—thereby underscoring the novelty and diversity of the solution set established in the present work.

**Table 6:** Comparison between the types of exact solutions obtained in the current work and those reported in Ref.^28^.

Type of the obtained solutions	The current work	Ref.^28^
Composite hyperbolic type	Exist, (Eq. (38))	Exist
Dark soliton	Exist, (Eq. (40))	Not Exist
Singular periodic	Exist, (Eq.’s (39), (41))	Exist
Exponential traveling wave	Exist, (Eq. (42))	Not Exist
Hyperbolic wave	Exist, (Eq. (43))	Not Exist
Singular hyperbolic soliton	Exist, (Eq. (47))	Not Exist
Singular mixed-type soliton	Exist, (Eq. (51))	Not Exist
Hyperbolic soliton	Exist, (Eq. (58))	Not Exist
Periodic trigonometric wave	Exist, (Eq. (59))	Exist
Kink-type	Exist, (Eq. (42))	Not Exist
Jacobi elliptic waves	Exist, (Eq.’s (62–82))	Not Exist
Bright soliton	Exist, (Eq. (66))	Exist
Solitary wave	Exist, (Eq. (78))	Exist

## Conclusion

This study shows that the Modified Extended Mapping (Mod–EM) method, applied to Loss–NLETL models with conformable spatial derivatives, generates a broad set of exact, physically interpretable solutions, hyperbolic and trigonometric waves, dark solitons, exponential travelers, singular and mixed kink–rational forms, and Jacobi-elliptic patterns. The results clarify how fractional parameters, especially the spatial order β₁ and dissipative terms control amplitude, width, and persistence, providing a systematic handle on waveform transitions and linking parameters to observable pulse behavior.

In comparison with other techniques, the Mod–EM method is particularly effective for non-integrable, dissipative, fractional systems. Hirota’s bilinear method and the Inverse Scattering Transform perform well for integrable, lossless equations but are less suitable in this setting. Reductive-perturbation approaches provide approximate envelope dynamics, whereas Mod–EM yields exact solutions for the full fractional, lossy model. Algebraic expansion schemes $\left(\mathrm{e}.\mathrm{g}.,\left({G}{\prime}/{G}^{2}\right)\right)$ can generate families of waves, but typically with a narrower scope. Moreover, Mod–EM complements Hamiltonian and bifurcation analyses by coupling explicit solutions with phase-plane structure, thereby clarifying parameter–stability relationships.

The results show that the Mod–EM method reliably generates a wide range of physically meaningful solutions for nonlinear, dissipative systems. The work deepens understanding of soliton behavior in electrical circuits. It also points to clear practical uses. These include pulse shaping and edge conditioning for high-speed electronics, better signal integrity in interconnects, and programmable dispersive delay lines for nonlinear filtering. The findings can also guide the design of switching networks and power-electronic devices where controlled energy localization under loss is crucial.

As natural extensions, the framework could be enriched by introducing external forcing or stochastic perturbations to study noise-induced soliton dynamics in realistic environments. Numerical simulations could validate analytical predictions, while alternative approaches such as generalized sine–Gordon expansions or Lie symmetry analysis might reveal new solution structures and hidden symmetries. Together, these directions would strengthen the connection between the analytical framework and engineering applications in nonlinear transmission line design and signal processing.

## Data Availability

The datasets used and/or analyzed during the current study are available from the corresponding author on reasonable request.
